# Artificial intelligence in ophthalmology: a bibliometric analysis of the 5-year trends in literature

**DOI:** 10.3389/fmed.2025.1580583

**Published:** 2025-07-01

**Authors:** Bosen Peng, Jiancheng Mu, Feng Xu, Wanyue Guo, Chuhuan Sun, Wei Fan

**Affiliations:** Department of Ophthalmology, West China Hospital, Sichuan University, Chengdu, China

**Keywords:** artificial intelligence, ophthalmology, diabetic retinopathy, glaucoma, machine learning, deep learning

## Abstract

**Purpose:**

This study aims to generate and elucidate the latest perspectives on the application of artificial intelligence (AI) in ophthalmology using bibliometric methods. By analyzing literature from the past 5 years (2020–2024), we seek to outline the development trends of this technology, provide guidance for its future directions, and assist clinicians in adapting to these innovations.

**Methods:**

We conducted a comprehensive search of all literature related to AI and ophthalmology in the Web of Science Core Collection (WoSCC) using bibliometric methods. The collected data were analyzed and visualized using three widely recognized bibliometric software tools: CiteSpace, VOSviewer, and the R package “Bibliometrix.”

**Results:**

A total of 21,725 documents were included from 134 countries and 7,126 institutions, consisting of 19,978 articles (91.96%) and 1,714 reviews (8.04%), with China and the United States leading the contributions. The number of publications in AI and ophthalmology has increased annually, with the University of California System, the National University of Singapore, and the University of London being the primary research institutions. Ophthalmology and Proc CVPR IEEE are the most co-cited journals and conferences in this field. These papers were authored by 87,695 individuals, with Wang Y, Liu Y, and Zhang Y the most prolific authors. Ting DSW was the most co-cited author. Major research topics include using various models to scan retinal images for diagnosing conditions such as age-related macular degeneration, diabetic retinopathy, and retinal nerve fiber layer thinning caused by glaucoma. The intersection of AI with other subfields of ophthalmology, such as in the diagnosis of ametropia, strabismus, eyelid disease, and orbital tumors, as well as in postoperative follow-up, is also rapidly developing. Key research hot spots are identified by keywords such as “deep learning,” “machine learning,” “convolutional neural network,” ”diabetic retinopathy,“ and ”ophthalmology.“

**Conclusion:**

Our bibliometric analysis outlines the dynamic evolution and structural relationships within the AI and ophthalmology field. In contrast to previous studies, our research transcends individual domains to offer a more comprehensive insight. Notably, our analysis encompasses literature published beyond the year 2022, a pivotal year marking both the post-pandemic era and the rapid advancement of AI technologies. This temporal scope potentially fills a gap that prior bibliometric studies have not addressed. This information identifies recent research frontiers and hot spot areas, providing valuable reference points for scholars engaging in future AI and ophthalmology studies.

## 1 Introduction

For Original Research Articles, Clinical Trial Articles, and Technology Reports, the introduction should be succinct, with no subheadings. For Case Reports, the Introduction should include symptoms at presentation, physical examinations, and laboratory results.

Artificial intelligence (AI) is now broadly used in many fields, especially in medical science. For example, machine learning such as basic regression and classification algorithms, decision trees and random forests, and support vector machines is widely used for predicting hypertension-supporting clinical decisions (1, 2). Deep learning, a subfield of machine learning inspired by the neural network structure of the human brain, is now used in the fields of processing images in medicine (3). This direction of AI, computer vision, is often utilized in the field of ophthalmology.

Ophthalmology has evolved from initial vision correction techniques to modern minimally invasive surgeries, with continuous advancements in technology and theory (4, 5). Historical milestones include the development of the ophthalmoscope and advancements in cataract surgery, which have greatly improved patient outcomes (6, 7). However, the field of ophthalmology still faces numerous challenges, such as early disease diagnosis, treatment outcome assessment, and patient-specific variations. These challenges drive researchers to explore new technological solutions to enhance the quality and efficiency of ophthalmic care. AI presents a promising avenue to address these challenges (8, 9).

The potential of AI in ophthalmology is vast, with applications ranging from automated image analysis to predictive modeling of disease progression. The intersection of ophthalmology with computer vision has emerged as a focal point of innovation, particularly in the realm of object detection (OD). Within this space, convolutional neural networks (CNNs, a class of deep neural networks designed to learn spatial hierarchies of features from input data using convolutional layers, pooling operations, and fully connected layers) stand out as prominent tools owing to their superiority in semantic segmentation and image analysis, making them indispensable in ophthalmic imaging. For instance, a notable study utilized this algorithm to sample every pixel on the corneal limbus, calculating the distance from the pupil center to the inner and outer canthus, thereby facilitating the screening of strabismus (10). Another research endeavor applied the same algorithm to scan 245,760 images to assess the thickness and density of the meibomian glands, a critical factor in diagnosing meibomian gland dysfunction (11). Additionally, a variety of models, such as ResNet, AlexNet, VGGNet, and the Inception series, have been effectively employed in diagnosing retinal diseases (12–15). Beyond computer vision, the integration of AI in ophthalmology has also extended to natural language processing. Recurrent neural network models such as Graph LSTM have shown remarkable prowess in utilizing diverse datasets, including electronic health records (EHR) and optical coherence tomography (OCT, a non-invasive imaging modality that uses low-coherence interferometry to generate high-resolution, cross-sectional images of biological tissues in real time) images, for the diagnosis of ocular diseases (16). Moreover, there have been studies proposing the use of multimodal integration models for the treatment of pulmonary infections, indicating potential applicability within ophthalmology as well (17).

The convergence of AI with the field of ophthalmology has garnered increasing attention in recent years. This fusion presents numerous opportunities for advancements in clinical practices, diagnostic accuracy, and patient outcomes. Given the rapid development and integration of AI technologies, a bibliometric analysis in this area is not only timely but also essential. While a number of bibliometric studies concentrating on AI and ophthalmology were published in 2022, they primarily focused on literature up to 2021 or were confined to specific subfields within ophthalmology (18, 19). For example, studies conducted by Monson et al. and Tang et al. only focus on literature from 2018 to 2021 (20, 21). In addition, the research proposed by Zhang et al. and Zhou et al. only focuses on thyroid-associated ophthalmopathy or strabismus, which lacks a comprehensive understanding of the combination of AI and ophthalmology (22, 23). In comparison, our study not only pays attention to literature beyond 2021 but also discusses the integration of AI and ophthalmology from a comprehensible perspective. This analysis pinpoints 2022 as a pivotal year, not just due to the end of the COVID-19 pandemic, but also because it marked significant advances such as the emergence and widespread adoption of ChatGPT and large language models. These developments have introduced unique progress and shifts that earlier studies might not have captured comprehensively, limiting their relevance for future research trajectories. In this study, we employ three tools to conduct a comprehensive bibliometric study and visualization analysis of the literature from 2020 to 2024. Our focus specifically targets articles indexed within WoSCC concerning the application of AI in ophthalmology. We believe that this methodological framework allows us to encapsulate a broader range of the latest developments and applications of AI models in the field of ophthalmology, providing an in-depth perspective on the promising future roles AI is poised to play in ophthalmic practices.

## 2 Materials and methods

### 2.1 Search strategies and data collection

**Figure 1:**
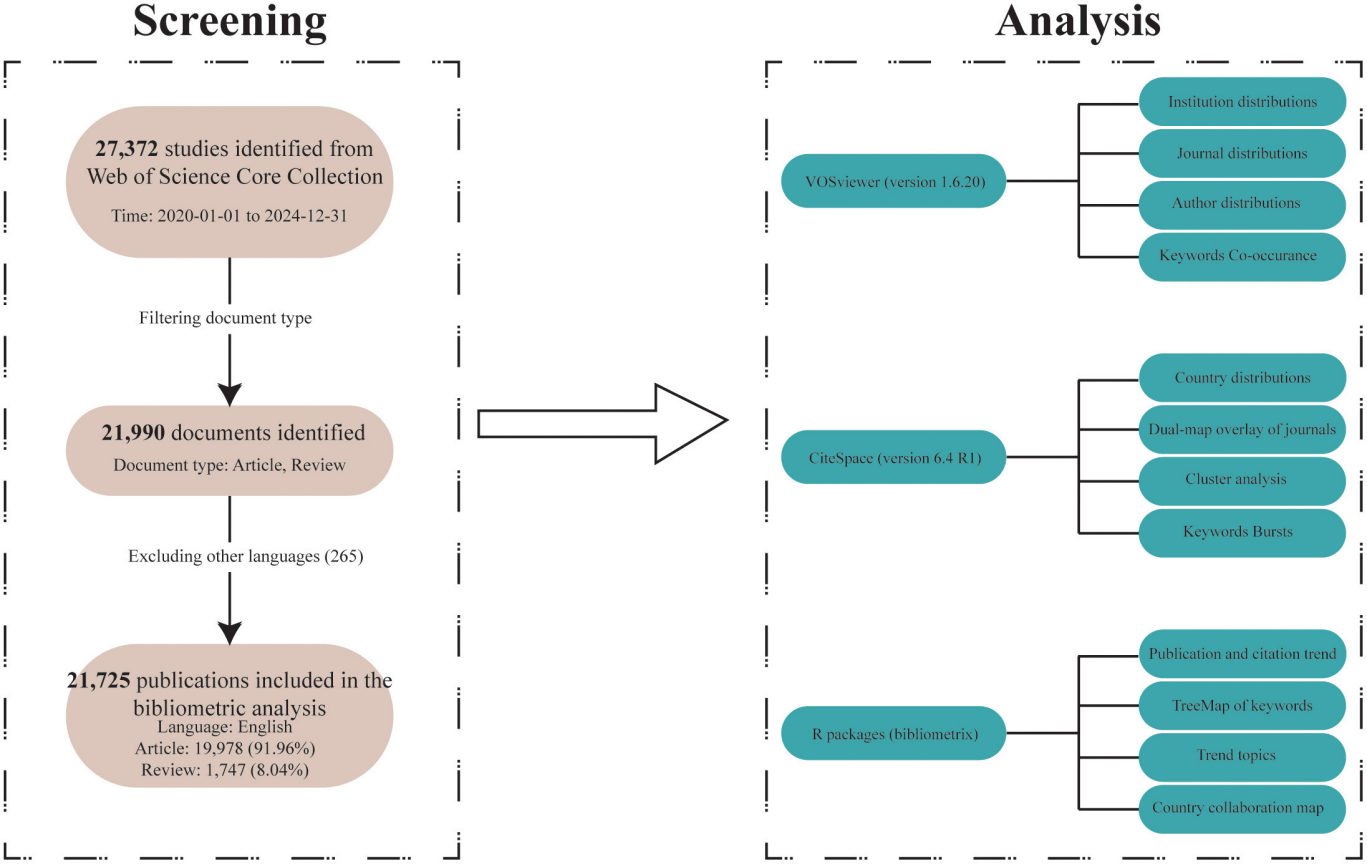
The flow chat of literature screening and analysis in the bibliometric study.

Since the analytical tools we used only support analyzing data from one database format at a time, and it is nearly impossible to merge and reanalyze results from different databases, we could only select data from a single database for analysis. Among PubMed, Scopus, Embase, and Web of Science, we chose Web of Science, which is a relatively authoritative database that includes high-quality research from both the medical and computer science fields. Furthermore, to ensure the quality of the included studies, we limited our analysis to literature from the Web of Science Core Collection (WoSCC, https://webofscience.clarivate.cn/wos/woscc/advanced-search) to reduce bias caused by potential variations in research quality. We identified all relevant literature on AI and ophthalmology from that database. Our search was conducted on May 30, 2025 (This is to avoid data bias from subsequent literature updates). Publications containing terms related to ”artificial intelligence“ and ”ophthalmology“ in their titles, abstracts, or keyword lists were deemed eligible. The specific search strategy applied can be seen in Table 1. Through this strategy, we identified 21,990 records from WoSCC. In our study, English-language publications dominated the retrieval results. In addition, multilingual articles may lead to inconsistencies due to language differences and limited software support for non-English texts. The inclusion of those could affect the accuracy of keyword extraction, co-word analysis, and topic clustering. Therefore, we excluded 265 non-English articles. Our bibliometric analysis incorporated 21,725 publications, comprising 19,978 articles and 1,747 reviews (Figure 1). Eligible records were saved and exported as plain text files that included titles, authors, keywords, institutions, countries, publication journals, references, and citations.

**Table 1:** Specific search strategy to obtain data from Web of Science Core Collection.

**Order**	**Search**
#1	TS=(artificial intelligence^*^) OR TS=(deep learning) OR TS=(machine learning) OR TS=(CNN^*^) OR TS=(RNN^*^) OR TS=(AI) OR TS=(network) OR TS=(image cognition)
#2	(TS=(ophthalmology) OR TS=(eye^*^)OR TS=(orbit) OR TS=(conjunctiva) OR TS=(sclera) OR TS=(cornea) OR TS=(lens) OR TS=(vitreous) OR TS=(uvea) OR TS=(choroid) OR TS=(retina^*^) OR TS=(macula) OR TS=(optic disk)) OR TS=(eye disease) OR TS=(ocular disease) OR TS=(Keratoconus) OR TS=(dry eye) OR TS=(glaucoma) OR TS=(myopia) OR TS=(cataract) OR TS=(retinopathy) OR TS=(fundus image) OR TS=(eyelid tumor)
#3	#1 and #2
Document types	Article and Review Article
Time span	From 2020-01-01 to 2024-12-31
Language	English

### 2.2 Data analysis

#### 2.2.1 VOSviewer

We employed VOSviewer (version 1.6.20), a bibliometric analysis software, to extract key information from the vast number of retrieved publications (24, 25). VOSviewer is typically used to construct networks for collaboration, co-citation, and co-occurrence (26, 27). In this study, the software facilitated several analyses, which included the following:

1. Country and Institution Analysis: Identification of countries and institutions contributing to AI and ophthalmology research.
2. Journal and Co-cited Journal Analysis: Examination of the publication and the citation landscape to understand the impact and relevance of different journals.
3. Author and Co-cited Author Analysis: Mapping the influential researchers and their citation networks.
4. Keyword Co-occurrence Analysis: Identifying prevalent research themes and trends.

In the network visualizations produced by VOSviewer, each node represents an item such as a country, research institution, journal, or author. The size and color of the nodes indicate the number and category of these items, respectively. The thickness of the lines connecting nodes reflects the extent of collaboration or co-citation among them.

#### 2.2.2 CiteSpace

CiteSpace (version 6.4 R1), developed by Professor Chen C., was utilized for further bibliometric analysis and visualization (28–31). This software enabled us to create dual-map overlays of journals, providing insights into the interdisciplinary nature and citation patterns of the research (32–35). Additionally, we leveraged the Citation Burst feature to identify references that have attracted rapidly increasing attention over specific periods.

#### 2.2.3 Language R

The R package “bibliometrix” (version 4.3.0) (https://www.bibliometrix.org/home/) was employed to perform thematic analysis and construct a global distribution network of AI and ophthalmology-related publications (36–41). Journal rankings and impact factors were sourced from the Journal Citation Reports 2022. Moreover, Microsoft Office Excel 2019 was used to conduct a quantitative analysis of the published literature.

VOSviewer is adept at generating network visualizations in a clear and concise manner, while CiteSpace, in addition to network mapping, highlights nodes with betweenness centrality. However, its visualizations tend to be more cluttered, making it a valuable complement to VOSviewer. Moreover, CiteSpace is better suited for tasks such as dual-map overlay analysis, time-series clustering, and burst detection. The R package “bibliometrix,” on the other hand, focuses more on analyzing the data itself, such as co-occurrence frequencies, citation counts, and trend patterns. In our study, we integrated these tools; when multiple tools could perform the same type of analysis, we carefully weighed their respective strengths and weaknesses, striving to balance the clarity of the visualizations with the depth of information provided while minimizing redundancy and contradictions in the results. For example, when analyzing journals in a related field, we used VOSviewer to generate the network map, CiteSpace for dual-map overlay visualization, and R to analyze co-citation frequencies, among other metrics. By integrating various analytical tools, our study may offer a comprehensive understanding of the research landscape in AI and ophthalmology, identifying key trends, influential contributors, and collaborative networks.

## 3 Results

### 3.1 Analysis of publication and citation

**Figure 2:**
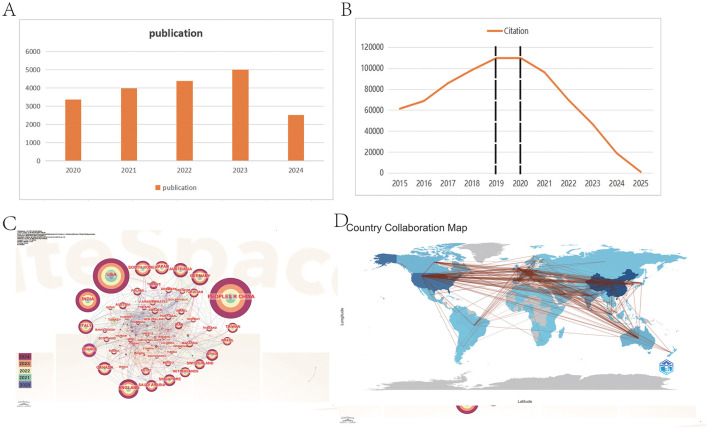
Distribution and trend of publications and citations from different years and countries. **(A)** The report of publication in 5 years. **(B)** The citation trend from 2015 to 2025. **(C)** The visualization of cooperation between countries. **(D)** The geographical distribution of publication. From CiteSpace 6.4 R1 and R package “Bibliometrix.”

The volume of published and cited literature is indicative of the speed, quality, innovation, maturity, and contextual factors surrounding the development of research in a specific field (42). In this study, we observed a significant upward trend in the publication of research combining ophthalmology and AI over the past 5 years, as illustrated in Figure 2A. Although the publication volume for 2025 is currently low, it can be predicted that it will surpass the figures for 2024 as research in this year continues to expand.

Moreover, the citation trend over the past decade (Figure 2B) can be categorized into three distinct phases: Phase 1 (2015–2018), Phase 2 (2019–2020), and Phase 3 (2021–2025). During Phase 1, the annual citation figures rose sharply from 61,311 to 109,462, nearly doubling. This surge correlates with the establishment of OpenAI in 2015, reflecting a growing focus among researchers on AI development and its applications in ophthalmology (43). In contrast, Phase 2 saw citation counts plateauing at approximately 110,000 annually, likely impacted by the COVID-19 pandemic, which hampered research activity in this domain (44–46).

Interestingly, Phase 3 showcases a declining citation trend despite an ongoing increase in publication output. This paradox may be attributed to the lag characteristic of citation cycles; following the transformative advent of AI technologies such as ChatGPT and advanced deep learning techniques since 2021, earlier AI literature may have become less appealing to researchers, resulting in decreased citation rates (47–50). Moreover, this phenomenon may also be attributed to the saturation of AI applications in disease recognition. For instance, in the diagnosis of diabetic retinopathy (DR, a complication of diabetes that affects the blood vessels in the retina, the light-sensitive tissue at the back of the eye), there has been an exponential increase in AI-related models and studies in recent years. This surge has resulted in an accumulation of low-quality and repetitive research, which, in turn, has diluted the impact of truly valuable contributions and led to a decline in citation rates. Furthermore, we posit that this trend may reflect an urgent demand for AI-driven research in ophthalmology, which has drawn a substantial influx of researchers into the field. Nonetheless, this decline is likely temporary and is typical in new or emerging fields, as it often takes time for new literature to gain traction in subsequent research.

Overall, these findings underscore that the intersection of AI and ophthalmology is a burgeoning field engaging numerous researchers, with promising future growth and potential.

### 3.2 Country and institution analysis

In our analysis of country and institution, we identified a total of 134 countries and 7,126 institutions contribute to the publication. Table 2 presents the top 10 countries and institutions that have made substantial contributions to the literature in this domain.

**Table 2:** Top 10 countries and institutions on research of AI and ophthalmology.

**Rank**	**Country**	**Counts (%)**	**Institution**	**Counts (%)**
1	China (Asia)	22,561 (27.3%)	University of California System	1,086 (1.2%)
2	The United States (North American)	15,243 (18.5%)	University of London	1,019 (1.1%)
3	India (Asia)	5,250 (6.4%)	National University of Singapore	935 (1.0%)
4	United Kindom (Europe)	3,997 (4.8%)	Chinese Academy of Sciences	872 (0.9%)
5	South Korea (Asia)	2,975 (3.6%)	Harvard University	858 (0.9%)
6	Germany (Europe)	2,765 (3.3%)	University College London	772 (0.8%)
7	Italy (Europe)	2,632 (3.2%)	Fudan University	665 (0.7%)
8	Japan (Asia)	2,277 (2.8%)	Shanghai Jiao Tong University	651 (0.7%)
9	Canada (North American)	2,092 (2.5%)	Sun Yat-sen University	638 (0.7%)
10	Australia (Australia)	2,042 (2.5%)	Harvard University Medical Affiliates	586 (0.6%)

The predominant countries contributing to the publications are primarily located in Asia, North America, and Europe. Notably, China leads with the highest number of publications (*n* = 22,561, 27.3%), followed by the United States (*n* = 15,243, 18.5%), India (*n* = 5,250, 6.4%), the United Kingdom (*n* = 3,997, 4.8%), and South Korea (*n* = 2,975, 3.6%). The combined number of publications from China, the United States, and India accounts for more than half of the total publications (52.2%). We further filtered our data to visualize 70 countries with the minimum number of publications equal to 5, constructing collaboration networks based on their cooperative relationships and citation data, as illustrated in Figures 2C, D. The results highlight active international collaborations, such as the strong connections between China and countries such as the United States, Japan, and Singapore, as well as interactions between the United States and France, Australia, and England. Interestingly, despite their relatively low publication volumes, France has been denoted with purple circles, indicating higher centrality (an indicator of research impact) in the network. It suggests that their research remains closely aligned with the core themes of this field and connects various sections within it.

**Figure 3:**
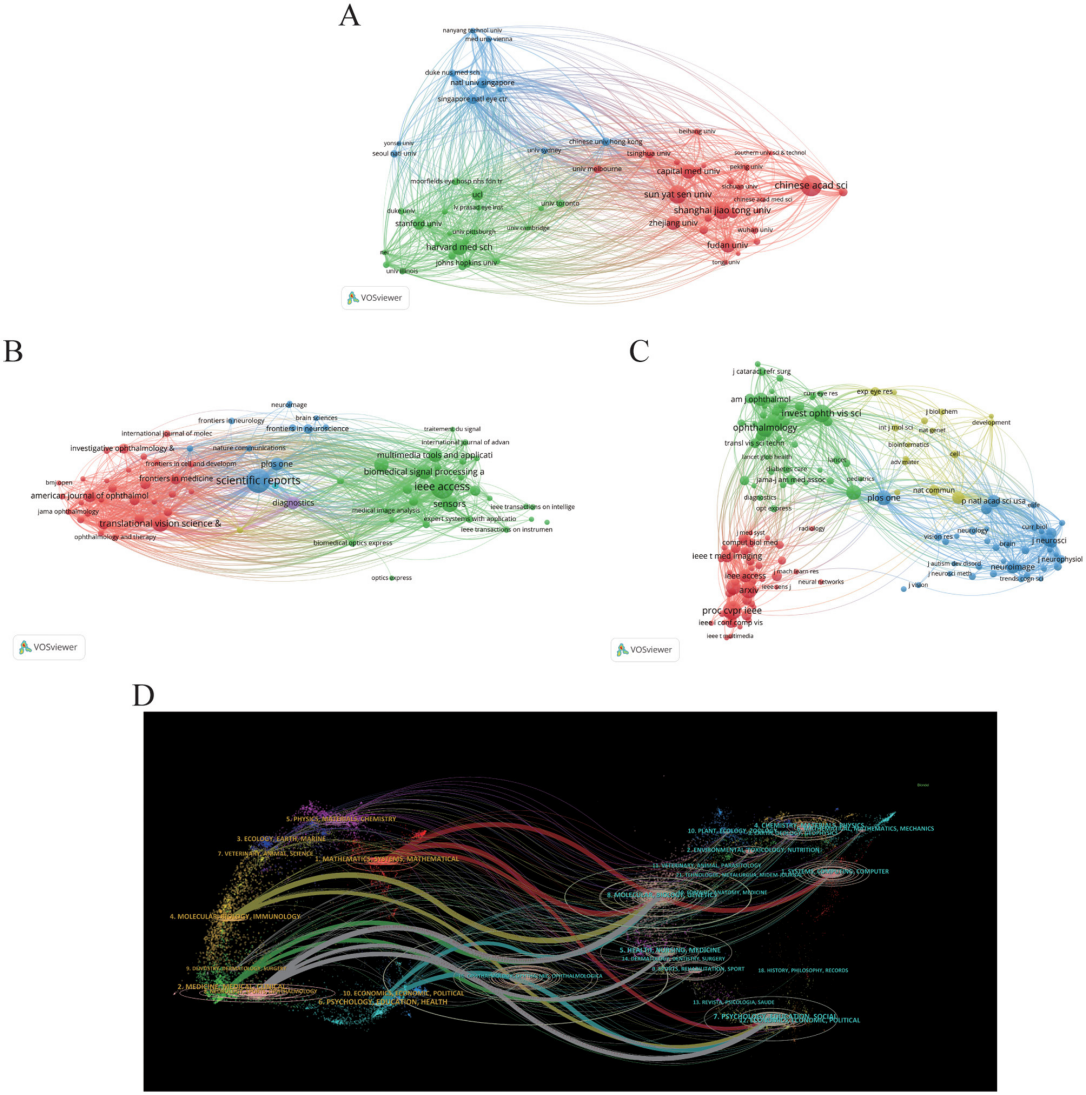
Distribution of publications and citations from different institutions and journals. Visualization maps of the institution **(A)**, journals **(B)**, and co-cited journals **(C)**. Institutions or journals with more publications or higher co-citation frequency are symbolized as the larger nodes. **(D)** The dual-map overlay of journals reveals the connections between publications and citations, with dots representing citing journals on the left and cited journals on the right so that the citation relationships are depicted as colored lines from the left to right. From: VOSviewer and CiteSpace 6.4 R1 Advance.

The majority of the top 10 institutions contributing to this field are located in China and the United States. The leading institutions include the following: the University of California System (*n* = 1,086, 1.2%), the University of London (*n* = 1,019, 1.1%), the National University of Singapore (*n* = 935, 1.0%), the Chinese Academy of Sciences (*n* = 872, 0.9%), and Harvard University (*n* = 858, 0.9%). We visualized collaboration among 58 institutions with a minimum publication volume of 100, depicted in Figure 3A. The analysis reveals significant collaborations, such as between Sun Yat-sen University in China and the Singapore National Eye Centre and Shanghai Jiao Tong University. Additionally, the National University of Singapore maintains active ties with Stanford University and Harvard University, with a notable closeness in collaboration between Stanford and Harvard as well.

### 3.3 Journals and co-cited journals

In our study, we identified that a total of 4,076 journals related to the application of AI in the field of ophthalmology have been published across various journals. Among these, Scientific Reports leads with the highest number of related articles (*n* = 577, 2.7%). Following closely are IEEE Access (*n* = 556, 2.6%), Translational Vision Science & Technology (*n* = 324, 1.5%), and Sensors (*n* = 316, 1.5%), as illustrated in Table 3. This distribution of publications across well-known journals highlights the growing integration of ophthalmology and AI, particularly where computer science meets medical sciences. To better understand the interconnectivity of these journals, we filtered the results based on a minimum publication threshold of 50 articles, ultimately analyzing 71 journals for their citation relationships. The resultant journal network visualization is depicted in Figure 3B, where it can be observed that Scientific Reports shares significant citation links with Translational Vision Science & Technology, IEEE Access, Sensors, and Multimedia Tools AND Applications, indicating collaborative research trends and thematic similarities among these publications.

**Table 3:** Top 10 journals and co-cited journals for research of AI and ophthalmology.

**Rank**	**Journal**	**Counts (%)**	**IF**	**Q**	**Co-cited journal**	**Citations**	**IF**	**Q**
1	Scientific Reports	577 (2.7%)	4.6	2	Proc CVPR IEEE	21,470	Conference	Conference
2	IEEE Access	556 (2.6%)	3.9	3	Invest Opth Vis Sci	19,509	Preprint Server	Preprint Server
3	Translational Vision Science & Technology	324 (1.5%)	3.9	3	Ophthalmology	19,441	13.7	1
4	Sensors	316 (1.5%)	3	3	Arxiv	15,246	4.4	2
5	Biomedical Signal Processing and Control	266 (1.2%)	5.1	2	PLoS ONE	12,787	2.9	3
6	Applied Sciences-Basel	248 (1.1%)	3.6	4	Sci Rep	12,723	4.6	2
7	Multimedia Tools and Applications	243 (1.1%)	2.7	4	Lect Notes Comput Sc	12,528	Conference	Conference
8	Plos One	216 (1.0%)	2.9	3	Am J Ophthalmol	9,867	4.1	1
9	Diagnostics	212 (1.0%)	3.6	3	IEEE Access	9,432	3.9	3
10	American Journal of Ophthalmology	204 (0.9%)	4.1	1	Neuroimage	9,124	4.7	2

Co-citation analysis further renders insight into the high-impact journals within this scholarly domain. In Table 2, the top 10 journals listed demonstrate citation counts exceeding 9,000. Notably, ophthalmology-related journals prominently feature in this list, while AI and computer science-related research is chiefly published in prestigious conference proceedings. This emphasizes both the distinctiveness and convergence of these fields. Among these journals, “Proc CVPR IEEE” is cited most frequently (21,470 times), followed by “Invest Opth Vis Sci” (19,509 times), “Ophthalmology” (19,441 times), and “arXiv” (15,246 times). For the visualization of the co-citation network, we filtered for a minimum co-citation count of 1,000, resulting in the analysis of 157 journals as illustrated in Figure 3C. In this visualization, the red clusters predominantly represent AI-related publication venues, while the green clusters are primarily from ophthalmology-focused journals, suggesting a vital intersection between these fields. The blue clusters represent the other medical fields related to ophthalmology where AI has also been applied. It can be seen that there is a positive co-citation relationship between ophthalmology journals represented by ophthalmology and AI journals represented by Proc CVPR IEEE.

It is also important to note that the Impact Factor (IF, a metric that reflects the average number of citations received per article published in a journal during the two preceding years) serves as a widely recognized indicator for assessing the core influence of journals. Among the 10 journals publishing related articles, the most influential is Biomedical Signal Proceeding and Control (IF = 5.1), followed by Scientific Reports (IF = 4.6). Within the co-citation analysis, “Ophthalmology” stands out as the highest impact journal (IF = 13.7) in the ophthalmology domain, with “American Journal of Ophthalmology” (IF = 4.2) ranking second. In contrast, “Proceedings of CVPR” remains a distinguished top-tier conference in the AI discipline.

Finally, the dual-map overlay of journals presented in Figure 3D illustrates the relationships between citing and co-cited journals. Clusters of citing journals are observed on the left side, while cited journals are clustered on the right, with citation relationships depicted via bold-colored lines emanating from left to right (29, 51). Seven principal lines are identified: three gray lines from Molecular/Nursing/Education categories to Neurology/Ophthalmology/Sports/Clinical, and three red lines from Computer/Molecular/Education to Mathematics/Systems/Ophthalmology. These pathways emphasize the intricate relationships among ophthalmology, medicine, mathematics, and computing, showcasing the interdisciplinary nature of current research trends. In addition, this analysis also highlights the application of computer science in basic medicine such as molecular and genetic study in ophthalmology. This analysis not only underscores the dynamic collaboration between ophthalmology and AI but also highlights the emerging trends and impact factors of academic publications in this evolving domain.

### 3.4 Authors and co-cited authors

In the field of AI application in ophthalmology, a total of 87,695 authors and 362,508 co-cited authors have made significant contributions, as presented in Table 4. Similar to the analysis of leading countries and institutions, the majority of these contributors hail from China, underscoring their leadership in this research domain.

**Table 4:** Top 10 authors and co-cited authors on reasearch of AI in ophthalmology.

**Rank**	**Author**	**Country**	**Counts**	**Co-cited author**	**Country**	**Citation**
1	Wang Y	China	194	Ting DSW	Singapore	1,367
2	Liu Y	China	177	Wong TY	Singapore	1,314
3	Zhang Y	China	175	Keane Pa	United Kindom	1,078
4	Zhang J	China	151	Zhang XL	China	944
5	Wang J	China	146	Fu HZ	China	900
6	Liu J	China	136	Cheung CY	China	898
7	Liu Y	China	134	Cheng CY	Singapore	862
8	Li J	China	120	Li F	China	760
9	Zhang L	China	108	Bogunovic H	Austria	739
10	Wang L	China	102	Liu Y	China	727

**Figure 4:**
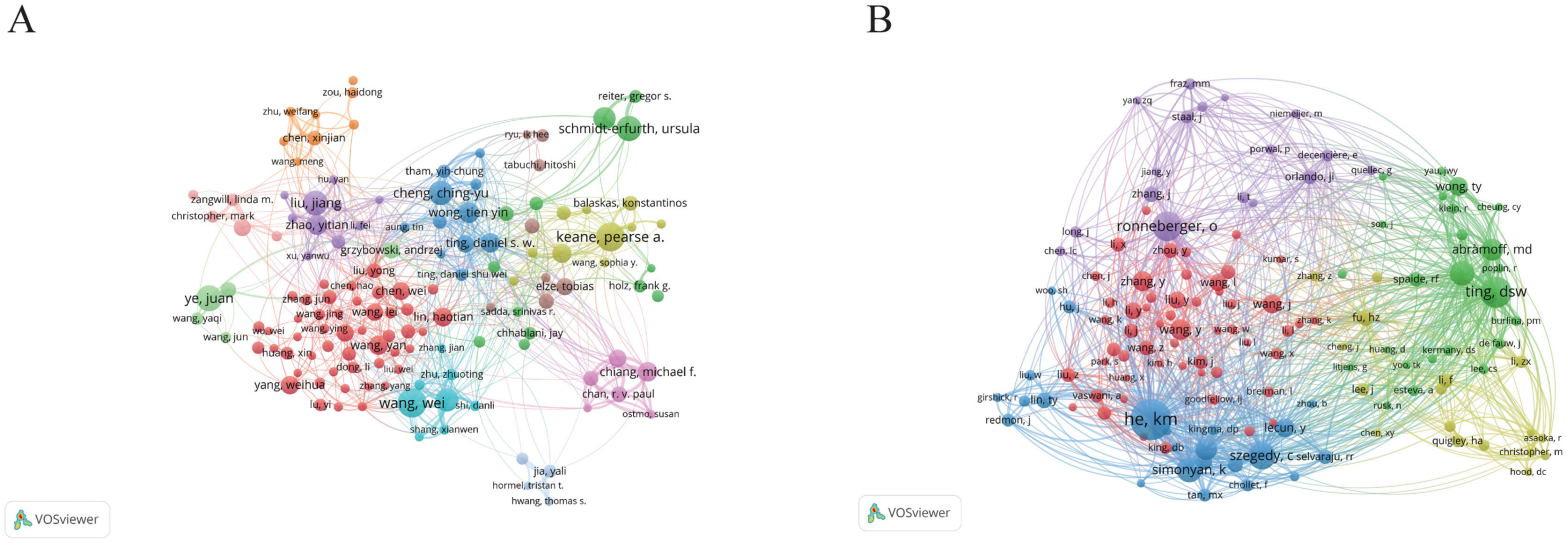
The visualization of authors **(A)** and co-cited authors **(B)** on research of AI in ophthalmology. From: VOSviewer.

The most prolific author is Wang Y, with 194 publications, followed closely by Liu Y with 177 and Zhang Y with 175. These authors are affiliated with prominent institutions, including Beijing Tongren Hospital, Zhongshan Ophthalmic Center, and Zhejiang University, respectively. Furthermore, we selected 136 authors with five or more publications to construct a collaboration network diagram (Figure 4A). Close collaborations among authors—such as Keane PA with Ting DSW and Lee with Campbell—are evident.

Among the top 10 co-cited authors, three authors have received over 1,000 citations. The most cited author is Ting DSW with 1,367 citations, followed by Wong TY with 1,314 and Keane Pa with 1,078. Notably, both Ting DSW and Wong TY are affiliated with the Singapore National Eye Centre, highlighting the prominent role of this institution in the integration of AI in ophthalmology research. Subsequently, we generated a co-citation network diagram for 136 authors with a minimum co-citation count of 300 (Figure 4B), revealing strong interconnections among various authors, such as Ting DSW, Wang TY, He KM, and Ronneberger O. This comprehensive analysis illustrates the collaborative nature and the influential authors shaping the landscape of AI in ophthalmology research.

### 3.5 Co-occurring keywords and burst terms

**Figure 5:**
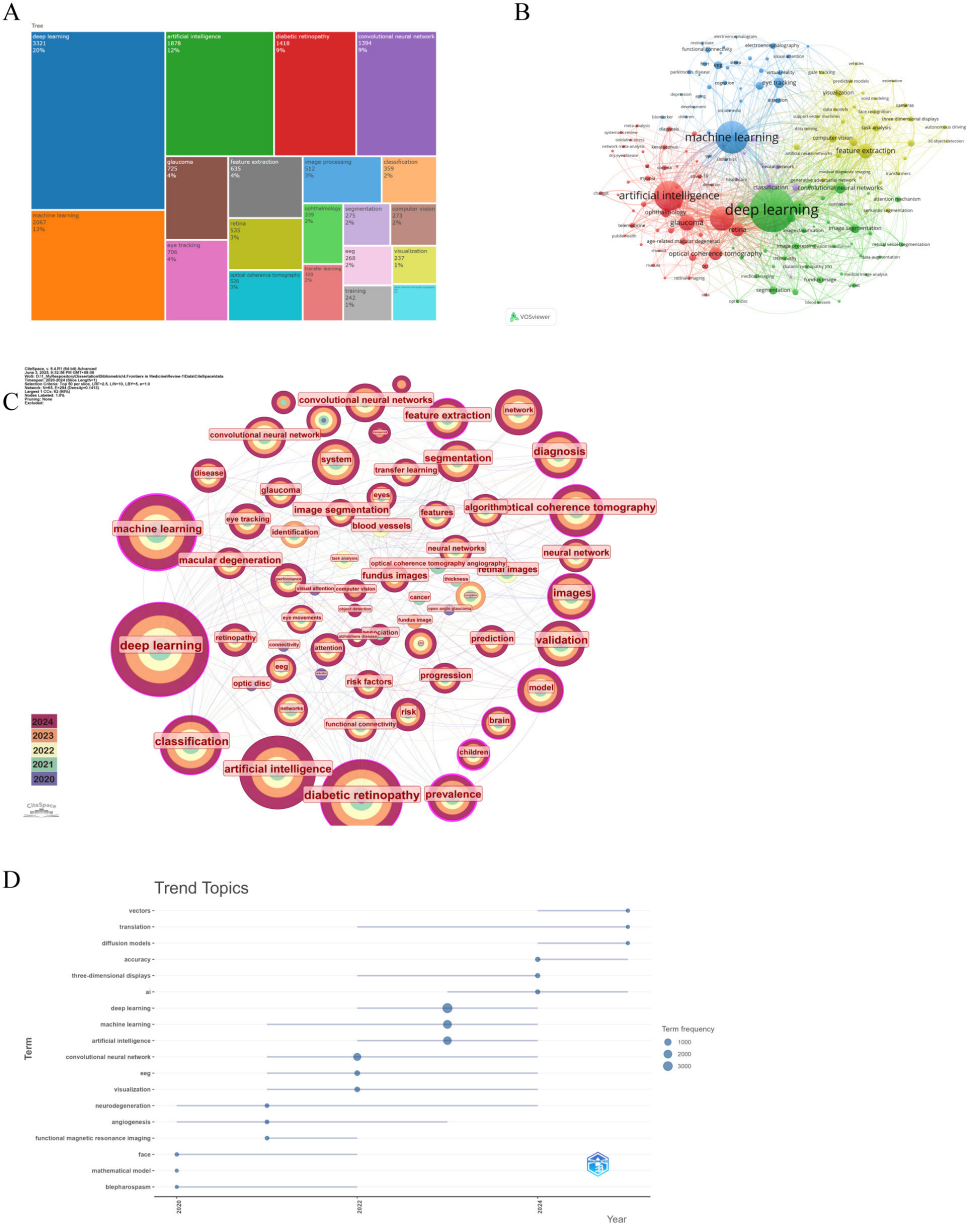
The main keywords. **(A)** The first 20 keywords belong to the publications. **(B)** Keywords co-occurrence networks. The node size indicates the frequency of keyword occurrence, and the lines connecting nodes represent the strength of the link between keywords. **(C)** Keywords distribution of the publications. Purple rings on the periphery mean a high centrality. **(D)** The trend topic analysis of keywords. From VOSviewer, CiteSpace 6.4 R1 Advance and R package “Bibliometrix.”

Through the co-occurrence analysis of keywords, we were able to quickly identify research hotspots within the field of AI applications in ophthalmology (52, 53). Similar or synonymous terms were merged for calculation (e.g., ”eye tracking“ and ”eye-tracking,“ ”convolutional neural network,“ ”cnn,“ and ”convolutional neural networks“). Table 5 lists the top 20 high-frequency keywords associated with AI in ophthalmology, which are also visually represented in the TreeMap Chart shown in Figure 5A. From this analysis, we can categorize the keywords into three main classes:

**Table 5:** Top 20 keywords on research of AI in ophthalmology.

**Rank**	**Keywords**	**Counts**	**Rank**	**Keywords**	**Counts**
1	Deep learning	3,321	11	Image processing	512
2	Machine learning	2,067	12	Classification	359
3	Artificial intelligence	1,878	13	Ophthalmology	339
4	Diabetic retinopathy	1,418	14	Transfer learning	320
5	Convolutional neural network	1,394	15	Segmentation	275
6	Glaucoma	725	16	Computer vision	273
7	Eye tracking	706	17	EEG	268
8	Feature extraction	635	18	Training	242
9	Retina	535	19	Visualization	237
10	Optical coherence tomography	526	20	Optical coherence tomography angiography	230

The first category comprises AI and computer science-related terms, which dominate the keyword list, including deep learning (*n* = 3,321, 20%), machine learning (*n* = 2,067, 13%), artificial intelligence (*n* = 1,878, 12%), convolutional neural network (*n* = 1,394, 9%), and computer vision (*n* = 273, 2%). The second category includes ophthalmology-related terms, such as diabetic retinopathy (*n* = 1,418, 9%), glaucoma (*n* = 725, 4%), and ophthalmology (*n* = 339, 2%). The third category consists of terms related to ophthalmic diagnostic technologies, such as eye tracking (*n* = 706, 4%) and optic coherence tomography (*n* = 526, 3%). Although this classification occupies a smaller portion of the keywords, it remains critical for highlighting significant tools and technologies in the field.

Based on keywords with a co-occurrence frequency of 50 or more, we selected 171 keywords to generate a co-occurrence network diagram using VOSviewer and CiteSpace, as depicted in Figures 5B, C. In Figure 5B, the blue and green sections mostly correspond to AI and computer science-related categories, while the red sections are mainly aligned with ophthalmology-related categories. The size of the nodes is positively correlated with their co-occurrence frequency, and the connecting lines depict their co-occurrence relationships. Notably, machine learning demonstrates a close connection with fundus, deep learning, diabetes retinopathy, and glaucoma, while deep learning shows a positive association with nearly all keywords. Additionally, in Figure 5C, the nodes for machine learning, deep learning, optic coherence tomography, and diabetes retinopathy are all annotated with purple rings, signifying their high centrality and reflecting their status as popular research keywords in the current landscape of AI applications in ophthalmology. Trend topic analysis (Figure 5D) indicates that before 2022, research on AI applications in ophthalmology primarily focused on mathematic models, angiogenesis, and blepharospasm to address various ocular diseases. In addition, the frequency of them is < 50. However, post-2022, following the advent of ChatGPT, the study of AI in ophthalmology surged. Therefore, research interest has shifted toward more advanced models such as convolutional neural networks, deep learning, and machine learning, and researchers try to develop more robust models with higher accuracy.

Through the analysis, we can identify the main focus of recent studies, which emphasize the application of AI technologies, such as deep learning and machine learning, in analyzing the results of ophthalmic diagnostic techniques, including fundus imaging and OCT, for diagnosing eye diseases such as DR, glaucoma, and myopia (54). Numerous cutting-edge studies have emerged in this field. For instance, Yoo et al. developed a deep learning model to predict uncorrected refractive errors in OCT images, preventing clinicians from overlooking refractive error-related risks during OCT assessments (55). Additionally, using OCT retinal images, Li et al. employed CNN models to identify pathological high myopia-related fundus lesions, such as macular holes, myopic choroidal neovascularization, and retinoschisis (56–58). Furthermore, researchers have utilized deep learning algorithms to enable AI to detect various characteristics in digital fundus images associated with DR, such as microaneurysms, hard exudates, and intra-retinal hemorrhages, achieving sensitivities of up to 50% (59). Lam et al. applied AlexNet, ResNet, and VGG16 techniques to identify DR lesions within image blocks, achieving an impressive accuracy of 98% based on 243 fundus images from EyePACS (60–62). In the realm of cataracts, Wu et al. collected data from 14,820 participants along with 16,200 fundus images of cataract and non-cataract cases, which were randomly divided into mutually exclusive groups for AI model training (development dataset) and internal testing (validation dataset). Following extensive validation, their model achieved an area under the curve (AUC, the area under the receiver operating characteristic curve, which measures the ability of a binary classifier to distinguish between positive and negative classes, with values ranging from 0 to 1) value of 99% during the quality testing phase (63–65). Collectively, these studies underscore the promising potential of AI applications in the field of ophthalmology.

### 3.6 Co-cited references

#### 3.6.1 Top cited references

References provide a substantial knowledge foundation and theoretical framework for subsequent scientific research. The citation frequency of references is crucial for any given article, reflecting its impact and relevance within the academic community. The underlying premise of co-citation analysis is that if two references are cited together by a single article, they are identified as exhibiting co-citation behavior, suggesting a strong relational context between the two. Consequently, a higher co-citation frequency indicates greater importance in the publication and a stronger guiding position within the field.

Table 6 presents the top 10 most cited references, showcasing a significant intersection between the domains of AI and ophthalmology. Notably, a conference publication by He et al. proposed a residual learning framework aimed at simplifying network training, subsequently enhancing the visual recognition capabilities of AI. This advancement is particularly influential in improving the diagnostic accuracy of diseases within ophthalmology (66). Furthermore, a prospective cohort study by Ting et al. primarily described the application of optical coherence tomography angiography (OCT-A) to quantify the retinal capillary microcirculatory system in DR patients, aiming to study diabetes and its complications (67). In the same year and in the same journal, Ting DSW developed and validated a deep learning system for DR and associated ocular diseases, showcasing impressive sensitivity and specificity (68). Additionally, De Fauw et al. integrated AI into their research, which involved training on 14,884 scans. Their findings illustrated that the suggestions of AI for retinal diseases surpassed those of experts across a range of vision-threatening retinal conditions (69). These references elucidate why they frequently appear in literature within this field.

**Table 6:** Top 10 co-cited references.

**Rank**	**Citation**	**Author**	**Reference title**	**Journal**	**Year**
1	2,004	He KM	Deep residual learning for image recognition	Proc CVPR IEEE	2016
2	1,678	Ronneberger O	U-net: Convolutional networks for biomedical image segmentation	Lect Notes Comput Sc	2015
3	1,254	Gulshan V	Development and validation of a deep learning algorithm for detection of diabetic retinopathy in retinal fundus photographs	JAMA-J AM	2016
4	1,219	Simonyan K	Very deep convolutional networks for large-scale image recognition	Arxiv	2015
5	1,063	Krizhevsky A	ImageNet classification with deep convolutional neural networks	Commun ACM	2017
6	814	Ting DSW	Development and validation of a deep learning system for diabetic retinopathy and related eye diseases using retinal images from multiethnic populations with diabetes	JAMA-J AM	2017
7	665	Deng J	Imagenet: A large-scale hierarchical image database	Proc CVPR IEEE	2009
8	601	Huang G	Reducing the memory cost of training convolutional neural networks by CPU Offloading	Proc CVPR IEEE	2017
9	552	Szegedy C	Rethinking the inception architecture for computer vision	Proc CVPR IEEE	2016
10	542	Staal J	Ridge-based vessel segmentation in color images of the retina	IEEE T Med Imaging	2004

Subsequently, we selected 231 references based on a co-citation frequency of 100 or more, as depicted in Supplementary Figure 1A. The green and yellow clusters represent references related to AI and computer science, while the red cluster pertains to ophthalmology. The visualization clearly identifies the interconnections between the two disciplines, highlighted by the close co-citation relationships among the 2016 paper published in Proc CVPR IEEE by HE, the contribution from the same year in JAMA by Gulshan, and the 2017 article published in JAMA by Ting DSW. This interrelationship underscores the collaborative evolution of AI technologies and their application in ophthalmological studies, emphasizing the growing synergy between these two critical areas.

#### 3.6.2 Reference with citation burst

References with citation bursts typically indicate that specific studies have been frequently cited by scholars within a defined timeframe. A total of 25 references exhibiting strong citation bursts were identified by CiteSpace. As shown in Supplementary Figure 1B, each band represents a specific year, with the red bands indicating years demonstrating significant citation burstiness.

Before 2022, the references predominantly focused on image recognition related to deep learning and retinal diseases, particularly DR. Among these, the reference with the highest citation burst strength was the study conducted by Gulshan et al., published in 2016 in JAMA-J AM (”Development and validation of a deep learning algorithm for detection of DR in retinal fundus photographs“) with a strength of 126.23. In this pivotal study, the researchers compared the performance of a deep learning algorithm in identifying DR from retinal fundus photographs with the manual grading accuracy of ophthalmologists. Using a dataset of 9,963 images for training and 1,748 images for validation, they reported a sensitivity of 90.3% and 87.0% and a specificity of 98.1% and 98.5%, respectively (70). The study emphasized that although the sensitivity and specificity of the deep learning algorithm were comparable to those of ophthalmologists, the technological advantages lay in the speed of diagnosis and the absence of fatigue associated with long-term assessments. The second strongest citation burst was from He et al., whose paper published in 2016 in Proc CVPR IEEE (”Deep Residual Learning for Image Recognition“) achieved a strength of 124.54. This reference introduced new techniques aimed at enhancing the visual recognition capabilities of AI, underscoring its significant implications within the field of ophthalmology due to both its high citation volume and intensity of citation bursts (66).

Post-2022, our observations indicate a shift toward more complex new technologies built upon deep learning frameworks, including conditional adversarial networks, pyramid scene parsing networks, and squeeze-and-excitation networks (71–75). Additionally, the scope of diseases being addressed expanded beyond just DR to encompass a wider array of ophthalmic conditions such as pathological myopia, glaucoma, and cataracts (76–78). Among these developments, the study of Orlando et al. published in 2020 in Ophthalmology, titled ”REFUGE Challenge: A unified framework for evaluating automated methods for glaucoma assessment from fundus photographs“ garnered noticeable attention with a citation burst strength of 20.69. The researchers of this study primarily launched the Retinal Fundus Glaucoma Challenge and released a large-scale glaucoma-related database. In addition, the study established a unified evaluation framework, enabling standardized and fair protocols for comparing different algorithms. The research found that the best approach for glaucoma classification combines deep learning techniques with well-known glaucoma-specific biomarkers, such as changes in the vertical cup-to-disc ratio or defects in the retinal nerve fiber layer. Automated methods using fundus imaging showed promising signs in identifying glaucoma suspects. For the segmentation task, the best solutions took into account domain shifts between the training and test sets, aiming to regularize models to handle image variations. The most challenging cases involved blurred boundaries between the optic disc and the optic cup. Further studies are needed to improve outcomes in these situations (79).

#### 3.6.3 Ten clusters of the co-citation network and cluster dependencies

The log-likelihood ratio algorithm is a powerful tool for efficiently processing large-scale datasets and analyzing data with multiple dimensional features. This algorithm is commonly applied across various fields for classification and model selection challenges. In our study, we employed CiteSpace to categorize the references into distinct clusters, each of which represents a sub-topic. The definitions of these sub-topics stem from the title terms of the cited papers within each cluster.

Supplementary Figure 1C illustrates the top 15 clusters identified in our analysis. The clusters are enumerated as follows: #0 artificial intelligence, #1 retinal vessel segmentation, #2 3D object detection, #3 ChatGPT, #4 diabetic retinopathy, #5 convolutional neural network, #6 diabetes, #7 classification, #8 retinal vessels, #9 image segmentation #10 glaucoma, #11 retinal nerve fiber layer, #12 feature extraction, #13 machine learning, and #14 age-related macular degeneration. Each of these clusters encompasses a body of reference focused on specific themes relevant to the application of AI in ophthalmology, thereby reflecting the current trends and research interests in these areas.

This analytical framework not only elucidates the thematic structure of literature but also underscores the collaborative nature of research scholarship in the intersection of ophthalmology and AI. Thus, the findings contribute to a comprehensive perspective on the advancements being made within this rapidly evolving field.

#### 3.6.4 Timeline map of clusters

The clustering analysis yields significant insights into the temporal evolution and development of various research areas within the field of ophthalmology, illustrated adeptly through a timeline graph (Supplementary Figure 1D). This graph effectively transforms the clustering map into a chronological representation, capturing the emergence of each cluster. As highlighted in previous discussions of cluster dependencies, the timeline vividly portrays the interconnections between different clusters and references over time, with each line connecting every node indicating the relationships between these clusters.

The earliest clusters identified in our analysis are convolutional neural network (Cluster #5), retinal vessels (Cluster #8), image segmentation (Cluster #9), and glaucoma (Cluster #10). These clusters signify an initial interest in developing efficient AI methodologies within the ophthalmological domain, laying the groundwork for later advancements in algorithmic types, such as retinal vessel segmentation (Cluster #1), 3D object detection (Cluster #2), and ChatGPT (Cluster #3). Concurrently, two additional clusters emerged in the same timeframe: diabetic retinopathy (Cluster #4) and retinal vessel segmentation (Cluster #1). Although these clusters exhibited limited integration with AI concepts at that stage, their early attempts to bridge both fields foreshadowed their subsequent high centrality (denoted by purple rings) and citation frequency (represented by large nodes) in future literature.

Despite the profound impact of ChatGPT and its accompanying large language models on the ophthalmic landscape, the presence of references pertaining to ChatGPT is currently minimal, and the inter-cluster relationships appear less than favorable. We hypothesize that this lagging citation cycle contributes to the observed pattern. Nonetheless, we predict a future spike in centrality and citation frequency for this cluster. Currently, the utilization of AI in ophthalmology predominantly focuses on retinal imaging, encompassing diabetic retinopathy (Cluster #4), retinal vessels (Cluster #8), and glaucoma (Cluster #10), in which AI is used to evaluate the thickness of the retinal nerve fiber layer (Cluster #11).

#### 3.6.5 Paper with high betweenness centrality

In the timeline visualization, several nodes are highlighted with purple circles, indicating that these references exhibit high betweenness centrality. This suggests that such references play a pivotal role as bridges between various research directions within the field of ophthalmology and AI. By facilitating the flow of knowledge, these key references connect otherwise disparate lines of inquiry.

Among the 15 clusters identified, 38 references were with notably high betweenness centrality. We summarize the top 10 in Table 7. Notably, within the cluster about glaucoma (Clusters #10 and #11), six references marked with purple circles are present. This underscores the significant role these references play in linking AI applications to ophthalmology, particularly in the context of glaucoma research (79–84). Moreover, the #13 cluster (machine learning) and the #8 cluster (retinal vessels) each feature two references highlighted with a purple circle, all of which are also related to retinal conditions (68, 70, 85, 86). This trend may be attributed to the earlier initiation of research in these areas, leading to rapid advancements in both ophthalmology and AI. Consequently, these clusters have stimulated further integration of AI technology across various subfields of ophthalmology. Additionally, there are other references with high betweenness centrality within clusters like artificial intelligence (#0), ChatGPT (#3), and diabetic retinopathy (#4). This phenomenon suggests that the interdisciplinary nature of the field has strong generalizability and has attracted concentrated attention from researchers.

**Table 7:** Top 6 highest “betweenness centrality” references among the 10 clusters.

**Rank**	**Centrality**	**References**	**Cluster #**
1	0.85	Development and validation of a deep learning system for diabetic retinopathy and related eye diseases using retinal images from multiethnic populations with diabetes	13
2	0.78	Development and validation of a deep learning algorithm for detection of diabetic retinopathy in retinal fundus photographs	13
3	0.76	REFUGE challenge: A unified framework for evaluating automated methods for glaucoma assessment from fundus photographs	10
4	0.75	Efficacy of a deep learning system for detecting glaucomatous optic neuropathy based on color fundus photographs	11
5	0.69	Deep convolution neural network for accurate diagnosis of glaucoma using digital fundus images	11
6	0.69	Performance of deep learning architectures and transfer learning for detecting glaucomatous optic neuropathy in fundus photographs	10
7	0.68	CNNs for automatic glaucoma assessment using fundus images: An extensive validation	10
8	0.67	Automatic glaucoma classification using color fundus images based on convolutional neural networks and transfer learning	10
9	0.50	DeepVessel: Retinal vessel segmentation via deep learning and conditional random field	8
10	0.48	Deep retinal image understanding	8

#### 3.6.6 Details of clusters #4 (diabetic retinopathy), #6 (diabetes), #1 (retinal vessel segmentation), #7 (classification), and #8 (retinal vessels)

In our cluster analysis, we identified the DR-related research areas: #4 (diabetic retinopathy), #6 (diabetes), #1 (retinal vessel segmentation), **#**7 (classification), and #8 (retinal vessels). By examining the timeline of these clusters, we considered factors such as betweenness centrality, citation impact, and publication year to select key references that offer a comprehensive understanding of the development of AI in the DR domain. The progress observed within these clusters reflects the gradual integration of AI technologies in the diagnosis and management of diabetic retinopathy (Table 8).

**Table 8:** The decades development of AI in fundus retinal disease (Clusters #0, #1, #2, #9) reflected in references.

**Clusters**	**2015**~**2019**		**2020**~**2024**	
	**Author (year), journal, volume**	**Citation**	**Author (year), journal, volume**	**Citation**
**#4 Diabetic retinopathy**				
	Wang SH (2018), Comput Electr Eng, 72	118	Huang SQ (2022), IEEE T Med Imaging, 41	31
	Zhang W (2019), Knowl-Based Syst, 175	88	Farag MM (2022), IEEE Access, 10	27
**#6 Diabetes**				
	Abràmoff MD (2016), Invest Ophth Vis Sci, 57	169	Dai L (2021), Nat Commun, 12	108
	Gargeya R (2017), Ophthalmology, 124	303		
**#7 Classification**				
	Qummar S (2019), IEEE Access, 7	164	Hemanth DJ (2020), Neural Comput Appl, 32	87
	Zheng XL (2019), IEEE Access, 7	78	Kalyani G (2023), Complex Intell Syst, 9	27
**#1 Retinal vessel segmentation**				
	Jin QG (2019), Knowl-Based Syst, 178	191	Chen J (2021), Arxiv, 0	72
	Gu ZW (2019), IEEE T Med Imaging, 38	214	Cao Hu (2023), Computer Vision-ECCV, 0	30
**#9 Retinal vessels**				
	Liskowski P (2016), IEEE T Med Imaging, 35	88	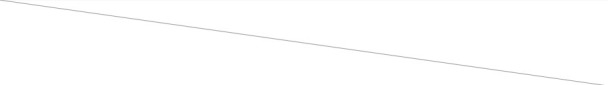	
	Li QL (2016), IEEE T Med Imaging, 35	67		

Between 2015 and 2019, Abràmoff et al. improved the automated detection of DR by integrating deep learning, using a consensus reference standard for referable diabetic retinopathy (rDR). DR was classified according to the International Clinical Diabetic Retinopathy Disease Severity Scale into moderate and severe non-proliferative DR (NPDR), proliferative DR, and/or macular edema (ME). Notably, the Messidor-2 images and the three retinal specialists who established the Messidor-2 reference standard were not involved in the training of the IDx-DR version X2.1. The study reported a sensitivity of 96.8%, a specificity of 87.0%, and an AUC of 0.98, indicating excellent performance (87). On the other hand, in 2017, Gargeya et al. built upon this foundation by training an automated identification model using 75,173 DR fundus images. Their method enabled easy visualization of abnormal regions through automatically generated heatmaps, which highlighted subregions of each input fundus image for further clinical review. This study achieved an AUC of 0.97, with sensitivity and specificity of 94% and 98%, respectively. These studies undoubtedly laid the groundwork for future model development and fostered the integration between the two fields (88).

Today, we witness remarkable advancements. With the increasing availability of data, improvements in AI technologies, and conscious efforts to bridge AI with ophthalmology, applications of AI have achieved significant improvements. Current AI systems surpass those of a decade ago in sensitivity, accuracy, false-positive rates, and even innovative functionality (89, 90). Moreover, AI's potential to improve healthcare delivery in low- and middle-income countries has become increasingly recognized. In 2022, Huang et al. introduced relational transformer blocks (RTBs)—composed of cross-attention and self-attention heads—to explore dependencies between lesions and other retinal tissues. Combined with a global transformer block, their method simultaneously segmented four DR lesions. This approach significantly outperformed methods from a decade ago, ranking first in AUC_ROC for EX, MA, and SE, and AUC_PR for EX and SE, and second in AUC_ROC and AUC_PR for HE (91). In another study, a model for automatic DR severity grading was developed based on DenseNet, convolutional block attention modules, and 13,000 annotated images. This method demonstrated robust performance and quality metrics while reducing spatial and temporal complexity. Additionally, a 2D Gaussian filter enhanced the quality of the fundus images. Finally, a weighted loss function using INS was constructed to address the class imbalance issue, thereby improving predictive performance across all categories (92).

Interestingly, since 2019, there has been a gap in references related to retinal vessels. We speculate that this cluster may have later merged with others, as from an ophthalmological perspective, it likely represents a subcomponent of broader DR-related research clusters.

#### 3.6.7 Details of clusters #10 (glaucoma), #11 (retinal nerve fiber layer), and #12 (age-related macular degeneration)

As shown in Supplementary Figure 1C, in contrast to DR—which exhibited prominent results in keyword analysis and reference citation bursts—our analysis of Clusters #10 (glaucoma), #11 (retinal nerve fiber layer), and #12 (age-related macular degeneration) revealed that most references with high betweenness centrality originated from Clusters #10 and #11. This suggests a strong integration between AI and glaucoma-related research and indicates that the models developed in this area are also applicable to other ophthalmic diseases, demonstrating high generalizability. On the other hand, the research timeline for age-related macular degeneration (AMD, a progressive eye disease that affects the macula, the central part of the retina responsible for sharp, central vision, which typically occurs in older adults and is a leading cause of vision loss in people aged 50 and older) is relatively short and includes few references with high betweenness centrality, implying that the field has not yet reached saturation and holds potential for further exploration. Similarly, as shown in Table 9, we selected several representative papers to illustrate the progress in this field.

**Table 9:** The decades development of semantic segmentation (#5) and oculomics (#8).

**Clusters**	**2015**~**2019**		**2020**~**2024**	
	**Author (year), journal, volume**	**Citation**	**Author (year), journal, volume**	**Citation**
**#10 Glaucoma**				
	Raghavendra U (2018), Inform Sciences, 441	125	Orlando JI (2020), Med Image Anal, 59	212
	Fu Z (2018), IEEE T Med Imaging, 37	197		
	Diaz-Pinto A (2019), Biomed Eng Oline, 18	118		
**#11 Retinal nerve fiber layer**				
	Christopher M (2018), Sci Rep, 8	102	Son J (2020), Ophthalmology, 85	71
	Li ZX (2018), Ophthalmology, 125	288		
	Liu HR (2019), JAMA Ophthalmol, 137	145		
**#14 Age-related macular degeneration**				
	Burlina PM (2017), JAMA Ophthalmol, 135	157	Khan SM (2021), Lancet Digit Health, 3	63
	Kermany DS (2018), Cell, 172	329	Tan TE (2021), Lancet Digit Health, 3	25

In the field of glaucoma, biological parameters such as the retinal nerve fiber layer (RNFL, the innermost layer of the retina, composed primarily of the unmyelinated axons of retinal ganglion cells) thickness are key indicators for evaluating retinal damage. One study developed a deep learning model for detecting glaucomatous optic neuropathy (GON, a progressive and characteristic form of optic nerve damage associated with glaucoma, typically resulting from retinal ganglion cell loss and corresponding axonal degeneration) based on 48,116 color fundus photographs. In this study, 21 trained ophthalmologists classified the images. GON was defined as a vertical cup-to-disc ratio of 0.7 or greater, along with other characteristic GON features. The deep learning system achieved an AUC of 0.986, with sensitivity of 95.6% and specificity of 92.0%. While the model delivered promising results, certain limitations remained: notably, false-negative and false-positive rates were still relatively high. False negatives were primarily due to pathological or high myopia, diabetic retinopathy, and AMD, while false positives often stemmed from physiological cupping (93). These shortcomings were likely due to algorithmic limitations at the time. As evidence of this hypothesis, Son et al. in 2020 developed a model capable of not only distinguishing glaucomatous from non-glaucomatous disc changes but also achieving an even higher AUC of 99.9% (94). Interestingly, after 2020, there has been a notable absence of glaucoma-related references. We propose two possible explanations: First, the field may have approached saturation; Second, in the integration of AI and ophthalmology, the research focus has shifted from single diseases to developing more generalized models applicable across various eye conditions. This trend is observable not only in glaucoma but also in DR and AMD, as evidenced by a decline in citation frequency and betweenness centrality in recent years. In contrast, Clusters #1, #7, and #12, though less studied in earlier years, have continued their timelines into recent years, suggesting growing interest and potential (95, 96).

In the AMD domain, one of the early influential studies was published in JAMA Ophthalmology. This research developed a deep convolutional neural network (DCNN, CNN with multiple layers of convolutional operations) model for the automated classification of AMD in color fundus photographs. The study compared this dedicated DCNN against alternative deep learning methods using transfer learning and general features, as well as trained human graders. The task was formulated as a binary classification problem—differentiating between no/early AMD and referable intermediate/late AMD. Multiple experiments were conducted using different data splits, involving over 130,000 de-identified images from 4,613 patients. The model's performance was evaluated against the gold standard from the NIH's Age-Related Eye Disease Study dataset, achieving an accuracy of 91.6% and an AUC of 0.95 (97). Although its performance was not outstanding compared to later models, it still outperformed contemporaneous methods using transfer learning, and despite falling short of expert graders in accuracy, its speed and scalability far exceeded that of human evaluation. Another landmark study, published in Cell, adapted the Inception V3 architecture using OCT images to guide anti-VEGF therapy decisions for AMD patients. The model demonstrated the ability to identify underlying pathology on tissue maps, enabling referral decisions and showing performance comparable to or even surpassing that of human experts. This supports timely diagnosis of conditions that may lead to irreversible severe vision loss (98). As the field evolved, Tan et al. developed a model using 226,686 retinal images to diagnose retinal diseases. Unlike earlier studies, this study employed a blockchain-based AI platform and incorporated data from multiple countries to improve generalizability. The model achieved an impressive AUC of 97.3%, marking a significant advancement in AI-assisted retinal diagnostics (99).

#### 3.6.8 Details of clusters #0 (artificial intelligence), #3 (ChatGPT), #5 (convolutional neural network), and #13 (machine learning)

The analysis of Clusters #0 (artificial intelligence), #3 (ChatGPT), #5 (convolutional neural network), and #13 (machine learning) reveals significant developments in the field of AI and computer science, particularly as they relate to applications in ophthalmology. By examining the references (Table 10) in the timelines of these clusters, we can glean insights into the evolution of these technologies and make predictions regarding the future trajectory of AI in the field of eye care.

**Table 10:** The decades development of AI in ophthalmology (Clusters #3, #4, #6) reflected in references.

**Clusters**	**2015**~**2019**		**2020**~**2024**	
	**Author (year), journal, volume**	**Citation**	**Author (year), journal, volume**	**Citation**
**#0 Artificial intelligence**				
	Abràmoff MD (2018), Npj Digit Med, 1	295	Xie YC (2020), Lancet Digit Health, 2	111
	De Fauw J (2018), Nat Med, 24	328	Teo ZL (2021), Ophthalmology, 128	157
	Schlegl T (2018), Ophthalmology, 125	150	Ruamviboonsuk P (2022), Lancet Digit Health, 4	56
**#3 ChatGPT**				
	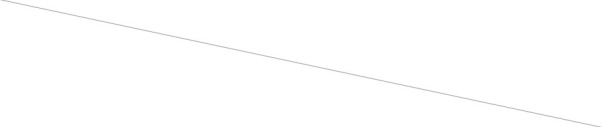		Antaki F (2023), Ophthalmol Sci, 3	39
			Mihalache A (2023), JAMA Ophthalmol, 141	31
			Momenaei B (2023), Ophthalmol Retina, 7	26
**#5 Convolutional neural network**				
	He KM (2016), Proc CVPR IEEE, 0	459	Wang QL (2020), Proc CVPR IEEE, 0	33
	Huang G (2017), Proc CVPR IEEE, 0	266		
	Krizhevsky A (2017), Commun Acm, 60	615		
**# Machine learning**				
	Gulshan V (2016), JAMA-J AM Med Assoc, 316	465	Sabanayagam C (2020), Lancet Digit Health, 2	26
	Ting DSW (2017), JAMA-J AM Med Assoc, 318	460	Zhang K (2021), Nat Biomed Eng, 5	24
	Krause J (2018), Ophthalmology, 125	142	Cheung CY (2021), Nat Biomed Eng, 5	53

In 2015, computer scientists began to re-evaluate the foundational architectures of computer vision, resulting in the introduction of the V3 version of the Inception architecture based on CNNs. This innovative framework broke down spatial representation into asymmetric, smaller convolutions, thereby reducing computational costs and effectively minimizing grid size. By employing label smoothing for model regularization, the framework achieved an impressive reduction in error rates for image recognition tasks, requiring merely 50 billion multiply-accumulate operations and containing fewer than 25 million parameters (100). Additionally, during conferences, researchers introduced the notion of leveraging deep residual learning to enhance image recognition efficiency (66). They pointed out future directions for AI development, such as representing feedforward networks as non-recurrent graphs extending from input layers to classifiers or regressors, and performing spatial aggregation within lower-dimensional embeddings (100, 101). At this stage, the attitude of ophthalmologists toward these AI advancements remained somewhat ambiguous, leading to a cautious approach to their application.

Currently, we observe the emergence of more complex and accurate models whose diagnostic capabilities often surpass those of clinical practitioners. Researchers have modernized the standard ResNet, steering it toward designs akin to vision transformers. Throughout this progression, several key components responsible for performance discrepancies were identified. A series of pure ConvNet models have been proposed, achieving a remarkable top-1 accuracy of 87.8% on ImageNet and demonstrating superior performance over Swin Transformers in COCO detection and ADE20K segmentation tasks, all while preserving the simplicity and efficiency characteristic of standard ConvNets (95). DenseNet has also utilized a method based on horizontal and vertical patch segmentation for preliminary experiments, yielding outstanding results (102). Zhou et al. introduced RETFound, a universal foundational model for disease detection from retinal images. RETFound can learn generalized representations from unannotated retinal images and provides a basis for label-efficient model adaptation across various applications. This approach offers a generalized solution to enhance model performance while alleviating the annotation workload for specialists, thereby facilitating widespread clinical AI applications in retinal imaging (103).

With the emergence of ChatGPT, an increasing number of researchers have begun to explore its potential integration into the field of ophthalmology. One notable study evaluated ChatGPT's performance on certification practice questions from the American Board of Ophthalmology. The results showed that ChatGPT correctly answered 58 out of 125 questions, yielding an accuracy of 46%. It performed best in the general medicine category (11/14; 79%) and worst in the retina and vitreous category (0%). The proportion of questions for which ChatGPT provided additional explanations was similar between correctly and incorrectly answered items. Additionally, the average length of both the questions and the answers was comparable between correct and incorrect responses. In 44% of the questions, ChatGPT's selected answer matched the most chosen option by ophthalmology residents on OphthoQuestions (*n* = 106). These results suggest that ChatGPT's medical knowledge may not yet be sufficiently reliable to support clinical use, and there is insufficient evidence to confirm its effectiveness in medical decision-making. It also reflects that we are still far from truly intelligent healthcare systems (104). As the study was conducted in January 2023, this may be attributed to ChatGPT being a newly launched model at that time, with limited intelligence and functionality. Another study reached similar conclusions. It generated two simulated examinations of 260 questions each, using the Basic and Clinical Science Course self-assessment program and the OphthoQuestions online database. These were used to test two versions of ChatGPT (the January 9 “legacy” model and ChatGPT Plus). Logistic regression was applied to assess how exam subject, cognitive level, and difficulty index influenced accuracy. The results showed that the legacy model achieved 55.8% accuracy on the BCSC set and 42.7% on the OphthoQuestions set. With ChatGPT Plus, accuracy improved to 59.4% and 49.2%, respectively. When controlling for exam section and cognitive level, questions with lower difficulty were more likely to be answered correctly (105). This study, proposed in December 2023, shows improved accuracy compared to the earlier study, suggesting ChatGPT has become more capable of handling complex ophthalmic clinical questions.

In contrast, the study by Momenaei et al. supports the clinical use of ChatGPT, although it emphasizes the need to inform patients, physicians, and laypersons about the limitations of these tools in ophthalmic and health-related consultations. The researchers compiled a list of common questions about retinal detachment (RD, an ophthalmic emergency in which the neurosensory retina separates from the underlying retinal pigment epithelium), macular hole (MH, a full-thickness defect in macula, specifically in the fovea, the area responsible for sharp central vision), and epiretinal membrane (ERM, a thin, fibrocellular layer that forms on the inner surface of the retina, particularly over the macula)—covering definitions, prevalence, visual impact, diagnostic methods, surgical and non-surgical treatment options, postoperative care, complications, and prognosis. Each question was asked three times on the ChatGPT-4 platform, and two independent retinal specialists rated the appropriateness of the responses. The results were promising: for RD, 84.6% (33/39) of responses were consistently correct, for MH, 92% (23/25) were consistently correct, and for ERM, 91.7% (22/24) were consistently correct. However, 5.1% (2/39) of RD, 8% (2/25) of MH, and 8.3% (2/24) of ERM responses were deemed inappropriate at least once. The Flesch–Kincaid grade levels and Flesch reading ease scores were as follows: RD with 14.1 ± 2.6 grade level and 32.3 ± 10.8 reading ease, MH with 14.0 ± 1.3 grade level and 34.4 ± 7.7 reading ease, as well as ERM with 14.8 ± 1.3 grade level and 28.1 ± 7.5 reading ease. These scores indicate that the responses were difficult to very difficult to read for the general public, requiring a college-level education to understand the content (106).

#### 3.6.9 Details of clusters #2 (3D object detection), #9 (image segmentation), and #12 (feature extraction)

The integration of ophthalmology and AI requires not only comprehensive data from the ophthalmic field and advanced models from computer science but also continuous improvements in image-processing technologies. Here, we also choose some typical references (Table 11) which can represent the advancement of this field. In 2018, Zhou et al. proposed VoxelNet in Proc. CVPR IEEE, a model designed for end-to-end 3D object detection based on point clouds. This model can directly process sparse 3D points and effectively capture 3D shape information (107). Although this technology was introduced relatively early, its integration with ophthalmology has so far been limited, with very few related publications. However, it still appears in citation clustering analysis, leading us to speculate that 3D object detection may become an important direction for future integration between the two fields. For instance, in cases where nodules or blebs develop following a trabeculectomy, this technology could potentially be used to analyze the scleral surface and help determine the underlying cause. One study proposed a novel method for constructing a 3D model of the scleral surface using light field image processing. First, a scleral surface light field imaging setup was developed using a Lytro Illum camera equipped with lighting. Second, the depth maps generated from the light field data were equalized and filtered to enhance grayscale quality. Third, edge detection information was added to extract texture features. Finally, 3D image reconstruction was performed. Based on the measurements, the average resolution of 3D images reconstructed from objects captured at a focal distance of 17 cm and a focal length of 80 mm was 0.14 mm. The study found that compared with other methods, image reconstruction using 3D object detection had lower error rates (108). Another study applied volumetric 3D scanning to detect vascular structures in fundus images. This model introduced two general-purpose curved object detectors, which can serve as building blocks for application-specific systems. These detectors used fuzzy mathematical morphological operators, which are robust against uncertainty and noise, and offer a balance between expressive power and computational demand. The extraction of linear features was based on fuzzy hit-or-miss transformation and fuzzy top-hat transformation, both of which can be customized according to the width of the target structures. Compared to other state-of-the-art general-purpose curved object detectors, this method was able to successfully locate the objects of interest across various grayscale images (109).

**Table 11:** The decades development of AI in keratoconus (#7).

**Cluster**	**2015**~**2019**		**2020**~**2024**	
	**Author (year), journal, volume**	**Citation**	**Author (year), journal, volume**	**Citation**
**#2 3D object detection**				
	Zhou Y (2018), Proc CVPR IEEE, 0	27	Dosovitskiy A (2021), Arxiv, 0	210
	Shi SS (2010), Proc CVPR IEEE, 0	23	Liu Z (2022), Proc CVPR IEEE, 0	45
**#9 Image segmentation**				
	Chen LC (2018), IEEE T Pattern Anal, 40	176	Tan MX (2021), Pr Mach Learn Res, 139	28
	Chen LCE (2018), Lect Notes Comput Sc, 11211	143		
**#12 Feature extraction**				
	Gulshan V (2019), JAMA Ophthalmol, 137	26	Bora A (2021), Lancet Digit Health, 3	24
	Schmidt-Erfurth U (2018), Prog Retin Eye Res, 67	193	Jin K (2022), Adv Ophthalmol Pract, 2	26

In the field of image segmentation, Ronneberger proposed a convolutional network designed for biomedical image segmentation in 2015. This image-processing strategy relies on powerful data augmentation techniques to make more efficient use of the available annotated samples. The proposed network architecture consists of a contracting path to capture context and a symmetric expanding path for precise localization. The study demonstrated that this network could be trained end-to-end with a very small number of images, and it outperformed the previous best method (sliding-window convolutional network) in the ISBI challenge for segmenting neuronal structures in electron microscopic stacks (110). Chen et al. proposed DeepLab, which leverages deep learning to address the semantic image segmentation problem. First, the study highlighted that convolutions with upsampling filters, or “dilated convolutions,” are a powerful tool for dense prediction tasks. Dilated convolutions allow the model to precisely control the resolution at which feature responses are computed within deep CNNs. They also expand the receptive field effectively, enabling the model to capture more context without increasing the number of parameters or computational cost. Second, the study introduced Atrous Spatial Pyramid Pooling for robust object segmentation at multiple scales. ASPP probes the input convolutional feature layer with filters at multiple sampling rates and effective receptive fields, enabling the model to capture both multiscale objects and contextual information within the image. Finally, the study improved object boundary localization by combining DCNN with probabilistic graphical models. While the commonly used max pooling and subsampling in DCNNs achieve invariance, they reduce localization accuracy. The researchers overcame this issue by combining the output from the final layer of the DCNN with a fully connected conditional random field, which significantly enhanced localization performance both qualitatively and quantitatively (111).

Finally, in the area of feature extraction, the VGGNet architecture has been widely used in the computer science community for extracting features from images, characterized by its uniform structure. One study utilized 76,370 retinal fundus images and combined two CNNs—an improved VGGNet architecture and a residual neural network architecture—to construct a model for classifying DR. The results showed that the AI system achieved an AUC of 0.973 (95% CI: 0.969–0.978) for referable diabetic retinopathy, with a sensitivity of 92.25% (90.10–94.12) and a specificity of 89.04% (87.85–90.28). The sensitivity for vision-threatening diabetic retinopathy was 99.42% (99.15–99.68), and for diabetic macular edema, it was 97.19% (96.61–97.77). The AI model showed comparable performance to human graders in detecting the prevalence of referable diabetic retinopathy and in identifying associated systemic risk factors. Both the AI model and human graders identified longer diabetes duration, higher HbA1c levels, and elevated systolic blood pressure as risk factors for referable DR (112). Additionally, Sayres et al. investigated the impact of a deep learning DR algorithm on physicians in a computer-aided environment. The study trained the model on 1,796 retinal fundus images from 1,612 diabetic patients and assessed DR severity based on the International Clinical Diabetic Retinopathy severity scale under three reading conditions: unaided, grade-only aid, and grade + heatmap aid. The grade-only aid condition included a histogram of DR predictions (grades) generated by the trained deep learning model. In the grade + heatmap condition, an explanatory heatmap was also provided. The results showed that under the grade-only condition, readers assisted by the model were more accurate than those without assistance. The addition of the heatmap further improved accuracy for DR-positive cases but reduced accuracy for DR-negative cases. Both aid conditions increased the sensitivity for detecting moderate or worse DR. These findings suggest that AI-assisted diagnosis improves the accuracy of retinal specialists, outperforming either unaided readers or the model alone. It also boosted readers' confidence and reduced reading time. In most cases, the grade + heatmap aid was as effective as the grade-only aid (113).

## 4 Discussion

### 4.1 Brief review of other applications of AI in ophthalmology

In our analysis, we found that the recent applications of AI in ophthalmology predominantly focus on image recognition, facilitating the diagnosis of eye diseases primarily related to retinal conditions such as DR, AMD, retinal vascular diseases, and changes in retinal nerve fiber layer thickness due to glaucoma. However, it is essential to recognize that the role of AI in ophthalmology extends beyond mere diagnostic support.

AI can significantly contribute to surgical assistance, predicting complications, and prognostication. Additionally, the predictive capabilities of AI can enhance preoperative assessments by identifying patients at higher risk for postoperative complications. Moreover, the potential applications of AI in ophthalmology are not confined to retinal diseases. Conditions affecting the anterior segment, such as refractive errors, cataracts, and corneal diseases, also stand to benefit from these advanced technologies. However, this area of application did not stand out in our previous bibliometric analysis. We speculate that this may be due to the following reasons: First, due to the current limitations of AI, both patients and clinicians still hold skepticism toward AI-driven medical decisions, which results in the small sample sizes in such studies (114–116). Second, these feature-rich AI models are relatively new developments in recent years, leading to a limited number of related publications. As a result, they have not yet been widely cited by other studies—though we believe this is a temporary phenomenon. As AI continues to evolve, it is evident that it can play a vital role across various subspecialties in ophthalmology. Therefore, a comprehensive review of AI applications in these broad areas is not only relevant but also crucial for understanding the future landscape of ophthalmic care and improving patient outcomes.

#### 4.1.1 The application of AI in hyperopia and astigmatism

Unlike myopia, where the spherical error is a critical predictive factor, the axial length is an essential parameter in hyperopia. Although the literature on AI applications specifically in hyperopia and astigmatism is sparse, we will provide an overview of notable advancements.

In the realm of hyperopia prediction and screening, a study developed a Classification and Regression Tree (CART, a type of decision tree algorithm used for both classification and regression tasks in machine learning) model to predict axial length based on spherical equivalent in hyperopia, for use when AL measurements are not available. The study divided hyperopic children into three age groups, measured and calculated the necessary parameters, and then used the CART model for analysis and prediction, comparing the results with a linear regression model. The study revealed that the CART model had an average absolute error of 0.60 when predicting AL across the three age groups, which was lower than that of the linear regression model (0.76) (117). Another notable study by Piotr et al. utilized a CNN to assess fundus images in hyperopic eyes. The performance of the model, when compared to two experienced ophthalmologists, achieved an accuracy of 93.3%, demonstrating high effectiveness. In intraoperative applications, Gupta et al. modeled the prediction of uncorrected visual acuity post-LASIK surgery for hyperopia. Their model successfully achieved a root mean square error (RMSE, a statistical measure used to evaluate the accuracy of a model's predictions) of 0.074 1 month postoperation, indicating robust predictive capability (118, 119). For prognostic purposes, Raychoudhury employed deep learning to develop a sensor-enhanced eyeglass named Activisee to detect discrete and continuous activities in presbyopic patients. This AI-driven device collects data for further intervention and effectively mitigates depth perception issues caused by multifocal lenses, thereby improving the safety and quality of life for presbyopic patients (120).

In astigmatism, the irregular curvature of the surface of the eye causes light rays to focus at multiple points, leading to blurred vision. The primary corrective approach for astigmatism is the use of rigid gas-permeable contact lenses. Hashemi et al. conducted a study to fit RGP lenses based on multi-view deep learning of Pentacam images. Utilizing CNN architectures such as AlexNet, GoogleNet, and ResNet, they identified features and performed transfer learning on 247 Pentacam refractive maps. Their results showed that the multi-view CNN achieved an R-squared value of approximately 0.83, indicating that this approach could expedite RGP lens fitting and enhance patient satisfaction (121). Another study used supervised image processing to classify Pentacam refractive maps and determine the base curve of RGP lenses for irregular astigmatism cases. By combining radial sector segmentation and deep CNN, the study succeeded in obtaining an R-squared value of 0.9642 and an RMSE of 0.0089. This technology significantly reduced trial-and-error during lens fitting and minimized patient visits (122). Wallerstein et al. applied deep learning to optimize intraocular lens (IOL, an artificial lens implanted inside the eye to replace the natural lens) calculators by predicting total corneal astigmatism (TCA, the amount of refractive error caused by the irregular curvature of the cornea) based on Pentacam tomographic data. Testing various regression learners, they found DNN most effective, significantly outperforming traditional methods in TCA magnitude prediction (*R*^2^ = 0.9740, RMSE = 0.0963 D, mean residual error = 0.0733 D) and reducing axis prediction error by an average of 8.1°. This advancement enables more precise TCA calculation and better IOL selection, potentially improving surgical outcomes (123).

In summary, while AI applications in hyperopia and astigmatism are still emerging, existing studies demonstrate promising results in prediction, screening, intraoperative support, and prognostic interventions. The integration of AI techniques such as CART, CNN, and DNN into ophthalmology offers significant potential for enhancing diagnostic accuracy, treatment precision, and patient outcomes.

#### 4.1.2 The application of AI in cataract

Cataracts, characterized by the clouding of the lens, are the leading cause of visual impairment globally. This condition accounts for approximately 51% of all blindness cases worldwide, with the majority found in developing countries (124, 125). The advent of AI holds significant promise in addressing and potentially mitigating this extensive public health issue.

Fan and colleagues undertook a pivotal study, amassing 647 high-quality images of cataracts spanning four stages. They employed a stratified random allocation method, dividing the data into a training set and a testing set at an 8:2 ratio to develop a classification model, and created both automatic and manual deep transfer learning (DTL, a machine learning technique that leverages the knowledge gained from a pre-trained deep neural network on one task and applies it to improve the performance of another related task) platforms. The results indicated that the automatic segmentation DTL platform achieved accuracies of 94.59% and 84.50% on the training and testing sets, respectively. In contrast, the manual segmentation DTL platform demonstrated accuracies of 97.48% and 90%. These findings suggest that automatic segmentation allows for quicker staging of cataracts, whereas manual segmentation offers higher accuracy. However, both models exhibited lower recognition rates for mature-stage images, often misidentifying them as overmature stages (54, 62). In terms of risk assessment, AI can be particularly useful in congenital cataracts by leveraging extensive genetic databases in conjunction with family history and lifestyle data—factors that traditional screening methods might overlook. This approach enables the prediction of cataract development in infants, allowing for continual monitoring and early intervention in high-risk populations (126). Furthermore, AI can analyze EHR to effectively identify at-risk patients in primary care settings, facilitating timely referrals to ophthalmic specialists (127).

AI has also been instrumental in enhancing the efficacy of cataract surgeries. Mohammadi and colleagues developed a prototype artificial neural network to accurately predict the posterior capsule status, thereby forecasting the occurrence of posterior capsule opacification post-phacoemulsification surgery. Utilizing the QUEST algorithm to construct decision trees, they developed three back-propagation artificial neural networks with 4, 20, and 40 neurons in two hidden layers, employing the same transfer functions (log-sigmoid and linear transfer) and training protocol. The optimal artificial neural network achieved an accuracy of 87%, compared to 80% for logistic regression (128, 129). Additionally, Kim et al. applied reinforcement learning to predict the relative location of the retinal surface to the current tool tip position, enhancing precision during ophthalmic surgeries. This method aids in reducing physiological tremors and providing accurate guidance for surgical maneuvers, particularly during intricate procedures like anterior capsulorhexis (116, 130).

In the aspect of following up, Edward and colleagues evaluated the accuracy and safety of a telemedicine call system, Dora R1, in detecting cataract surgery patients requiring further follow-up. The study involved 225 participants who received follow-up calls approximately 3 weeks post-surgery, supervised in real time by an ophthalmologist. The primary analysis compared the clinical significance of decisions made by Dora R1 and the supervising ophthalmologist concerning five distinct symptoms and the necessity for further examination. A secondary analysis used mixed methods to assess usability, acceptability, and cost impact relative to standard care of Dora R1. Dora R1 demonstrated 94% sensitivity and 86% specificity, showing moderate to high agreement with clinicians across all parameters. Additional studies on Dora corroborated these promising findings (131, 132).

The integration of AI and diagnosis, management, and postoperative care of cataracts presents a transformative potential in enhancing both the efficiency and accuracy of clinical practices. From improving diagnostic accuracy and risk assessment to optimizing surgical outcomes and facilitating postoperative follow-up, AI technologies are poised to significantly impact the global burden of cataracts, especially in resource-limited settings.

#### 4.1.3 The applications of AI in keratoconus

The integration of AI in the field of ophthalmology—particularly in the analysis and diagnosis of retinal diseases—is becoming increasingly prominent. Notably, although keratoconus did not stand out compared to other ophthalmic diseases in our previous bibliometric analysis, it is still reported in numerous research publications. This emerging trend suggests that AI is gradually expanding its application scope from retinal pathology to the anterior segment of the eye, thereby penetrating broader areas of ophthalmology.

In a highly significant study, researchers utilized tomographic data to enhance the sensitivity of detecting corneal ectasia. By generating an AI model based on Pentacam HR (Oculus, Wetzlar, Germany) parameters, they aimed to assess and detect corneal ectasia and keratoconus. The study conducted a comparative analysis of preoperative data from three patient groups: stable LASIK cases (2,980 patients with a minimum follow-up of 7 years), ectasia-susceptible cases (71 eyes from 45 patients who developed post-LASIK ectasia [PLE]), and clinically diagnosed keratoconus (182 patients). The model's accuracy was independently tested on another group of stable LASIK patients (298 patients, minimum 4-year follow-up) and 188 patients with very asymmetric ectasia—where one eye had a normal corneal topography, and the other had a clinical diagnosis of ectasia. The results showed that the Random Forest model achieved the highest accuracy. For clinically evident ectasia, it had a sensitivity of 100%, and across all cases, the AUC was 0.992 (sensitivity 94.2%, specificity 98.8%, cutoff 0.216). This performance was statistically superior to that of the Belin/Ambrósio deviation index (AUC = 0.960, sensitivity 87.3%, specificity 97.5%), indicating that the AI model significantly enhances the diagnosis of corneal ectasia. However, evaluating the risk of ectasia still requires integrating corneal biomechanical parameters and understanding the impact of laser vision correction procedures on corneal stability (133). In addition, Ghosh et al. conducted a study applying deep learning algorithms to rapidly differentiate between fungal keratitis (FK) and bacterial keratitis (BK). The study utilized a total of 2,167 anterior segment images from 194 patients, of which 128 had BK (1,388 images, 64.1%) and 66 had FK (779 images, 35.9%). The images were randomly divided into training, validation, and test sets, following two conditions: (1) The distribution of BK and FK was similar across all three datasets. (2) Each patient was assigned to only one dataset (no overlap). This resulted in a final split ratio of 85:5:10 for training, validation, and test sets, respectively. Three CNNs—VGG19, ResNet50, and DenseNet121—were trained to classify the images. The results showed the following classification performance: VGG19 reached an F1 score of 0.78, DenseNet121 of 0.71, and ResNet50 of 0.68. In terms of AUC: VGG19 achieved the highest AUPRC at 0.86, followed by ResNet50 at 0.73, and DenseNet121 at 0.60. Ensemble learning further improved performance, with a sensitivity of 0.77, an F1 score of 0.83, and an AUC of 0.904. These findings indicate that ensemble CNNs outperform individual models in distinguishing FK from BK, and the developed model holds promise as a supportive tool for rapid provisional diagnosis in patients with microbial keratitis (134–136).

Overall, the data substantiates the promising role of AI in advancing diagnostic accuracy for both anterior and posterior segment diseases in ophthalmology, paving the way for future innovations within this specialized domain.

#### 4.1.4 The applications of AI in other fields related to ophthalmology

Currently, the advantage of AI lies in its ability to handle large-scale data and provide more advanced and less error-prone statistical methods. Therefore, beyond improving classification accuracy and adding various functionalities in the field of image recognition through the use of multimodal data, AI advancements in ophthalmology are also actively transitioning into basic medical research and clinical applications.

In basic medical science, AI is often used to identify therapeutic targets for diseases. For example, Velez et al. used antibody microarray technology to analyze vitreous biopsy samples from patients with neovascular inflammatory vitreoretinopathy (NIV), detecting the expression of 200 cytokine signaling proteins. They compared the NIV samples at various stages of disease progression to those from non-NIV controls. Using simple unsupervised AI techniques such as hierarchical clustering and pathway analysis, they identified patterns in the data. Subjects treated with repositioning therapy were followed longitudinally. The model revealed key molecular pathways involved in NIV pathology, such as persistently elevated VEGF levels in mid-stage NIV patients, which correlated with disease progression—suggesting that anti-VEGF injections could alleviate vitreous hemorrhage without requiring vitrectomy. The study also identified mTOR and PI3K signaling pathways related to T-cell fate, implying that methotrexate could reduce NIV-specific T-cell inflammation without the side effects of corticosteroids. In addition, targeting IL-6 may help prevent recurrent fibrosis and retinal detachment (137). AI-driven models used for target identification share a data-driven learning foundation similar to those in drug development, leveraging diverse datasets to generate insights and predictions. Deep learning techniques such as DNNs and CNNs are commonly used in these models to capture complex patterns and relationships within the data. Tian et al. emphasized the importance of allosteric regulation in controlling protein activity, a critical aspect of drug development. Understanding allosteric mechanisms—especially the identification of allosteric sites—is a prerequisite for drug discovery and design. They proposed an ensemble learning approach that combines XGBoost and Graph convolutional neural networks to learn the physical properties and topology of protein binding pockets without prior knowledge. This method, embedded in tools like PASSer and CLI, aids in exploring protein allostery and drug development (138). Moreover, the integration of AI in ophthalmology-related bioinformatics is also gaining momentum. In combination with proteomics and metabolomics, one study measured 60 plasma cytokines in an initial cohort. In the validation cohort, ELISA kits were used to confirm six biomarkers: angiopoietin-1, CXCL16, platelet-derived growth factor-BB, TIMP-1, TIMP-2, and VEGF receptor 2. A machine learning algorithm was developed to build a predictive model for non-proliferative diabetic retinopathy. The results showed that plasma Ang-1, PDGF-BB, and VEGF-R2 were associated with the presence of NPDR, suggesting that these may serve as valuable biomarkers playing an important role in the pathophysiology of diabetic retinopathy (139).

In the area of AI-assisted clinical trials, Christopher et al. designed a deep learning–based model to improve the prediction of retinal structures and assist in glaucoma neuroprotection trials. The study included 3,327 paired GCIPL/RNFL scans from the macular posterior pole and ONH ring of 1,096 eyes from 550 patients, acquired using Spectralis. Participants were randomly assigned to training/validation datasets (90%) and a test dataset (10%). The network was given access to the GCIPL and RNFL data of the probed side of the retina, as well as all retinal data from the fellow eye. The model was trained to predict the contralateral GCIPL or RNFL thickness in the probed eye. The results showed the model could accurately predict superior and inferior GCIPL thickness, with global R^2^ values of 0.90 and 0.86, average R^2^ values of 0.90 and 0.86, and mean absolute errors (MAEs, a statistical measure used to evaluate the accuracy of a model's predictions) of 3.72 μm and 4.2 μm, respectively. For RNFL thickness prediction, the performance was slightly lower: global R^2^ values were 0.75 and 0.84, average R^2^ values were 0.81 and 0.82, and MAEs were 9.31 μm and 8.57 μm. When predicting GCIPL and RNFL in more advanced diseases, model performance declined only slightly. Using personalized hemiretina predictions to account for inter-patient variability, researchers estimated that a clinical trial could detect a 25% treatment effect over 24 months with 11 times fewer patients than traditional trials. This indicates the deep learning model can accurately estimate macular GCIPL and ONH RNFL hemi-retinal thickness, and model-based internal controls may help reduce sample size requirements, facilitating research into new glaucoma neuroprotective therapies (140). Another study explored the role of AI in clinical trial recruitment for geographic atrophy (GA, a specific type of degenerative change in the retina, particularly associated with AMD). Using OCT scan data, a deep learning model was trained to generate retinal tissue segmentation maps, identifying patients potentially eligible for GA trials. The effectiveness of this approach was compared to traditional keyword-based EHR searches. AI predictions were validated clinically using fundus autofluorescence (FAF, a non-invasive imaging technique used in ophthalmology to visualize the natural fluorescence emitted by certain structures within the retina) imaging and expert evaluation to calculate positive predictive value (PPV). The results showed that compared with the EHR search alone (PPV: 40%), the AI system achieved higher precision, with a PPV of 63% in identifying eligible patients. The combined AI-EHR method identified 604 eligible patients with a PPV of 86%. Among cases that met trial eligibility, the intraclass correlation between FAF-segmented GA areas and AI-segmented OCT areas was 0.77. The AI system also adapted to different imaging criteria across multiple trials, generating customized candidate lists ranging from 438 to 1,817 patients. This study demonstrated AI's potential to automate prescreening for GA clinical trials, including site feasibility assessment, data-driven protocol design, and cost reduction. Once therapies become available, similar AI systems may also be used to identify individuals likely to benefit from treatment (141).

AI also plays a significant role in Oculomics, a newly emerging multidisciplinary research field in ophthalmology. Oculomics involves analyzing ocular images and biomarkers to extract information about an individual's overall health and risk of systemic diseases. Poplin et al. used data from the UK Biobank and EyePACS databases, collecting retinal fundus images from over 280,000 patients to predict cardiovascular disease (CVD) risk factors. The model accurately predicted factors such as age and systolic blood pressure, with MAE of 3.26 years and 11.23 mmHg, respectively. It also achieved AUCs of 0.97 and 0.71 for predicting sex and smoking status, respectively. Furthermore, by directly associating retinal images with CVD events, the model predicted the risk of major adverse cardiovascular events (MACE) with an AUC of 0.70, comparable to the European SCORE risk calculator (AUC = 0.72) (142). Unlike Poplin's study, which was limited to cardiovascular risk factors, another research project developed deep learning algorithms to predict systemic biomarkers from retinal photographs. This study utilized 236,257 retinal images from seven different Asian and European cohorts, assessing the ability of 47 deep learning algorithms to predict 47 systemic biomarkers as outcome variables. These biomarkers included demographics (age and sex), body composition measurements, blood pressure, hematologic parameters, lipid profiles, biochemical indicators, and biomarkers related to liver function, thyroid function, renal function, inflammation, and diabetes. The results showed that the model could quantify body composition indicators (such as muscle mass, height, and weight) and creatinine from retinal photos. In the internal test set, the R^2^ for predicting muscle mass was 0.52, and in an external test set with actual muscle mass measurements, the R^2^ was 0.33. The R^2^ values for predicting height, weight, and creatinine in the internal test set were 0.42, 0.36, and 0.38, respectively. However, performance in external test sets, especially European cohorts, was considerably lower: R^2^ values for height ranged from 0.08 to 0.28, weight from 0.04 to 0.19, and creatinine from 0.01 to 0.26. Out of the 47 systemic biomarkers, 37 could not be well predicted from retinal images using deep learning (143). These studies exemplify the use of AI to explore the relationship between ophthalmology and systemic diseases, representing one of the promising directions for future development.

### 4.2 The challenges in the application of AI in ophthalmology

AI holds immense potential in the field of ophthalmology, but along with this promise come several pressing challenges. To achieve a successful transition and widespread adoption, we must address the obstacles that hinder its development.

#### 4.2.1 Data privacy and ethical issues

First and foremost is the issue of data privacy and ethics. With the extensive use of big data in the medical field, the importance of data privacy and ethical considerations has never been greater. To widely implement AI in ophthalmology, a vast amount of data from diverse patients across multiple regions is required for training. However, this kind of data sharing often involves sensitive information, which can violate regulations such as the General Data Protection Regulation in Europe and the Health Insurance Portability and Accountability Act in the United States (144). Additionally, data security, a critical aspect of data privacy, poses significant challenges. The aggregation of large amounts of sensitive data could become a single point of failure. Furthermore, healthcare data remains a frequent target for cyberattacks, particularly in Europe and the United States, where the healthcare sector continues to incur the highest average breach costs, exceeding $7 million (145, 146). Adversarial attacks can also exploit AI models by injecting compromised data during training (data poisoning) or altering input images, leading to widespread misclassification by the AI models (147, 148).

This issue has seen partial solutions, such as the adoption of federated learning, which allows researchers to train AI across institutions or borders without data sharing. Federated learning is a privacy-preserving technique exposing models to heterogeneous, non-independent, and identically distributed data (149). An extension called swarm learning can further decentralize AI model parameters, aiding in the development of generalizable models (150). Additionally, Generative Adversarial Networks can enhance these datasets, especially for rare diseases like congenital cataracts (151).

#### 4.2.2 Transparency and explainability of AI algorithms

The second challenge we face is the transparency and interpretability of AI algorithms. From a computational perspective, AI suffers from a ”black box“ phenomenon, where the decision-making processes are opaque and complicated (152–154). This lack of transparency and complexity makes AI tools difficult to manage and monitor, particularly in terms of accountability. Consequently, this contributes to patients' distrust toward AI in the ophthalmology field. Patients are less likely to opt for AI-based diagnostics if they are unsure about responsibility, diagnostic safety, and data privacy. On the other hand, doctors' empathetic abilities, which involve responding to patients' emotions, make patients more inclined to choose human doctors over AI (155, 156).

To address these concerns before integrating AI into clinical environments, future development should focus on enhancing policies that govern AI to clarify accountability and usage responsibilities. Technologically, solving the ”black box“ issue to improve transparency is crucial. Moreover, training should emphasize providing necessary humanistic care to patients (155).

#### 4.2.3 The accuracy of AI in ophthalmology

The third challenge is accuracy. Although many studies have highly praised AI in disease diagnosis, most of these were conducted on small sample sizes. Overfitting is a common issue resulting from the inherent problems in algorithm design. Overfitting occurs when artificial neural networks have too few samples or too many nodes, causing the model to learn random noise in the training data as concepts (155, 157). These concepts do not generalize to new validation data because they are highly tailored to the training data. Overfitting inflates accuracy and overestimates the clinical performance of the model. Accuracy can significantly drop when patient data, such as ethnicity or regional origin, is heterogeneous (153, 158, 159). This issue hinders AI deployment, suggesting that a crucial development direction is to expand datasets and use a more diverse population for AI training to improve generalizability. However, this also raises other ethical and privacy concerns (144, 160–162).

In conclusion, while AI holds tremendous promise in ophthalmology, addressing challenges related to data privacy and ethics, algorithm transparency and interpretability, and accuracy is vital for its successful integration and widespread adoption. Robust regulatory frameworks, technological advancements, and an emphasis on humanistic care will be essential in overcoming these obstacles.

### 4.3 Future prospects

At present, the integration of AI and ophthalmology is becoming increasingly close. However, as previously discussed, most existing studies are still focused on the classification and recognition of retina-related diseases. Although some research has attempted to apply AI to anterior segment areas of the eye, this field remains far from saturated. Future studies could incorporate more anterior segment data—such as slit-lamp examination, anterior segment OCT, and corneal topography—to explore ocular surface diseases, including eyelid-related disorders and corneoscleral diseases.

In addition, although AI has already made significant achievements in drug development and target identification, its application in ophthalmology remains limited. We speculate that this is partly because, compared to easily accessible ophthalmic imaging, obtaining drug- and protein-related data involves a longer cycle. On the other hand, computer science researchers and ocular basic science researchers may still lack awareness of the importance of interdisciplinary collaboration. Therefore, this area holds considerable potential for future exploration.

Finally, in terms of ethical AI deployment, it is essential not only to develop policies to protect data ethics and privacy but also to create more advanced encryption technologies to ensure data security during transmission and storage. In addition, continuous algorithm monitoring is needed. Developers should actively disclose algorithmic design approaches, training data, and testing results to enable external review and oversight. For instance, releasing white papers or technical reports can demonstrate algorithm transparency to users and regulators. To further promote AI deployment in ophthalmology, another crucial future direction is to define a clear responsibility framework and establish a liability insurance mechanism.

### 4.4 Advantages and shortcomings

This research possesses several unique advantages. First, we conducted a bibliometric analysis of the application of AI in the field of ophthalmology over recent years, particularly in the post-pandemic era and during the rapid development spurred by the advent of ChatGPT. A study conducted by Monson et al. lacks the use of CiteSpace. Therefore, they lack a deeper analysis such as burst citation analysis, cluster analysis, and timeline analysis, which results in that they only emphasize the application of AI in DR, while in our timeline analysis, we think glaucoma is also significant in this field (20). Tan et al. focus more on COVID-19-related telemedicine, as the time span of their analysis is from 2017 to 2021, which overlooks the application of large language models like ChatGPT in ophthalmology (21). Deng et al. also conducted a similar analysis. However, their study also lacks the analysis of cluster and timeline and therefore lacks the finding of other ophthalmic diseases and ChatGPT (18). Instead, our analysis provides a more comprehensive understanding for scholars focusing on this field, which previous studies may lack. Second, we utilized three bibliometric tools in our investigation, including VOSviewer and CiteSpace, which are widely used in the field of bibliometrics, thus ensuring the objectivity of our research. Finally, compared to traditional reviews, bibliometric analysis offers a more comprehensive view of the hotspots and frontiers in the field.

However, this study also has some shortcomings. First, as a consistent and standardized database, WoSCC ensures the feasibility of our research and the representativeness and high quality of our literature samples. In addition, due to the limitations of the tools we use, our data are sourced solely from WoSCC. Additionally, literature published after December 2024 was not included in this study, which is unavoidable, but we remain eager to observe the future integration of AI and ophthalmology.

## 5 Conclusion

In conclusion, AI holds significant research value and application prospects in ophthalmology. The rapid increase in publications indicates growing global interest, with China and the United States leading the way, while nations such as Singapore, India, and various European countries also make substantial contributions. However, enhanced collaboration among these countries and institutions is necessary. Articles and conferences in ophthalmology and computer vision are most prominent, showcasing the unique combination of these fields. Research on AI and retinal diseases remains a hotspot, likely making retinal applications the first AI integration in ophthalmology. Concurrently, the role of AI in anterior segment diseases such as refractive errors and cataracts is inevitable. Beyond diagnostics, AI will impact surgery planning, intraoperative assistance, and postoperative follow-up, simplifying data collection. Nonetheless, attention must be given to ethical and privacy concerns, and technical challenges must be addressed to accelerate clinical translation.

## Data Availability

The original contributions presented in the study are included in the article/Supplementary material, further inquiries can be directed to the corresponding author.

## Supplementary material

Supplementary Figure 1 The references. **(A)** The visualization of co-cited references on research of AI in ophthalmology. **(B)** The reference with citation burst by year. **(C)** CiteSpace visualization clusters of the co-cited references. Terms from the title field of the citing papers within each cluster are adopted as the definition of that cluster. **(D)** Timeline view of the listed clusters of the co-cited references. From: VOSviewer and CiteSpace 6.4 R1.
