# Supplementary figures and images for: Artificial intelligence in ophthalmology: a bibliometric analysis of the 5-year trends in literature
Source: Front Med (Lausanne). 2025 Jul 1;12:1580583. doi: 10.3389/fmed.2025.1580583 (PMC12259593; doi:10.3389/fmed.2025.1580583)

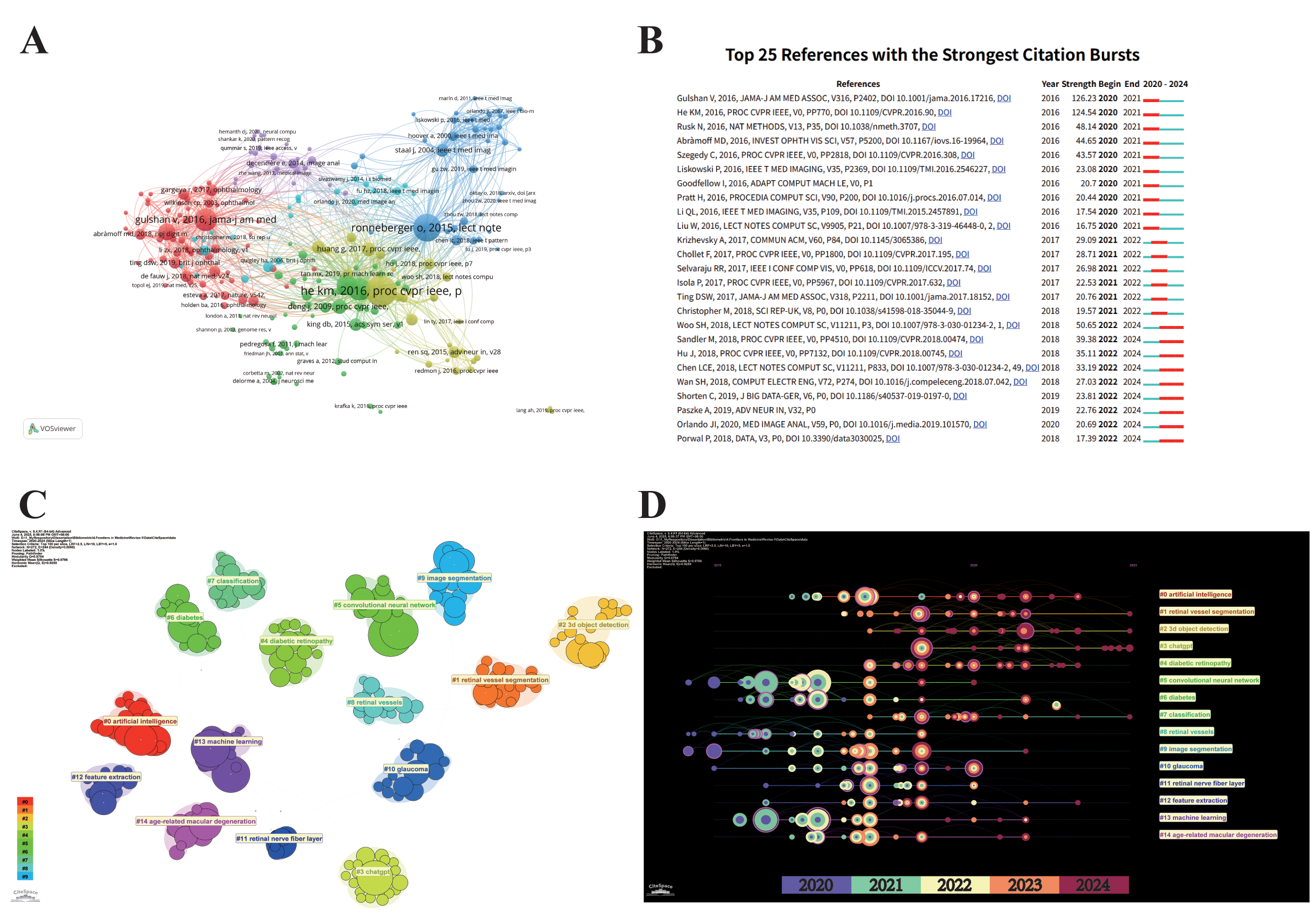

Supplement: Supplementary Figure 1 — The references. (A) The visualization of co-cited references on research of AI in ophthalmology. (B) The reference with citation burst by year. (C) CiteSpace visualization clusters of the co-cited references. Terms from the title field of the citing papers within each cluster are adopted as the definition of that cluster. (D) Timeline view of the listed clusters of the co-cited references. From: VOSviewer and CiteSpace 6.4 R1. [file Image_1.tif]
